# Restriction of *Francisella novicida* Genetic Diversity during Infection of the Vector Midgut

**DOI:** 10.1371/journal.ppat.1004499

**Published:** 2014-11-13

**Authors:** Kathryn E. Reif, Guy H. Palmer, David W. Crowder, Massaro W. Ueti, Susan M. Noh

**Affiliations:** 1 Department of Veterinary Microbiology and Pathology, Washington State University, Pullman, Washington, and Paul G. Allen School for Global Animal Health, Washington State University, Pullman, Washington, United States of America; 2 Animal Disease Research Unit, Agricultural Research Service, U.S. Department of Agriculture, Pullman, Washington, United States of America; 3 Department of Entomology, Washington State University, Pullman, Washington, United States of America; University of Texas at Austin, United States of America

## Abstract

The genetic diversity of pathogens, and interactions between genotypes, can strongly influence pathogen phenotypes such as transmissibility and virulence. For vector-borne pathogens, both mammalian hosts and arthropod vectors may limit pathogen genotypic diversity (number of unique genotypes circulating in an area) by preventing infection or transmission of particular genotypes. Mammalian hosts often act as “ecological filters” for pathogen diversity, where novel variants are frequently eliminated because of stochastic events or fitness costs. However, whether vectors can serve a similar role in limiting pathogen diversity is less clear. Here we show using *Francisella novicida* and a natural tick vector of *Francisella* spp. (*Dermacentor andersoni*), that the tick vector acted as a stronger ecological filter for pathogen diversity compared to the mammalian host. When both mice and ticks were exposed to mixtures of *F. novicida* genotypes, significantly fewer genotypes co-colonized ticks compared to mice. In both ticks and mice, increased genotypic diversity negatively affected the recovery of available genotypes. Competition among genotypes contributed to the reduction of diversity during infection of the tick midgut, as genotypes not recovered from tick midguts during mixed genotype infections were recovered from tick midguts during individual genotype infection. Mediated by stochastic and selective forces, pathogen genotype diversity was markedly reduced in the tick. We incorporated our experimental results into a model to demonstrate how vector population dynamics, especially vector-to-host ratio, strongly affected pathogen genotypic diversity in a population over time. Understanding pathogen genotypic population dynamics will aid in identification of the variables that most strongly affect pathogen transmission and disease ecology.

## Author Summary

Co-infection, the presence of multiple genotypes of the same pathogen species within an infected individual, is common. Genotype diversity, defined as the number of unique genotypes, and the interaction between genotypes, can strongly influence virulence and pathogen transmission. Understanding how genotypic diversity affects transmission of pathogens that naturally cycle among disparate hosts, such as vector-borne pathogens, is especially important as the capacity of the host and vector to sustain genotypic diversity may differ. To address this, we exposed *Dermacentor andersoni* ticks, via infected mice, to variably diverse populations of *Francisella novicida* genotypes. Interestingly, we found that ticks served as greater ecological filters for genotypic diversity compared to mice. This loss in genotypic diversity was due to both stochastic and selective forces. Based on these data and a model, we determined that high numbers of ticks in an environment support high genotypic diversity, while genotypic diversity will be lost rapidly in environments with low tick numbers. Together, these results provide evidence that vector population dynamics, vector-to-host ratios, and competition among pathogen genotypes play critical roles in the maintenance of pathogen genotypic diversity.

## Introduction

Genetic diversity within a single microbial species can lead to infection of hosts with mixtures of pathogen genotypes. Remarkably, studies across numerous systems have demonstrated that mixed-genotype infections are more common than infections with a single clonal variant [1]–[5]. The degree of genotypic diversity, defined here as the number of unique genotypes within a population, has been associated with pathogen transmission rates and virulence [6]–[9]. For example, greater numbers of circulating *Plasmodium faliciparum* genotypes were positively correlated with increased virulence or a greater probability of transmission [6],[8]. Competition experiments among Dengue virus serotypes resulted in the more virulent serotype being selected at the expense of less virulent serotypes during both human and mosquito infection [7]. Additionally, during the early years of West Nile virus circulation in New York, transmission intensity was associated with increases in viral genetic diversity [9].

The capacity of hosts to sustain multiple pathogen genotypes, and the within-host interactions among co-infecting genotypes, can impact pathogen transmission, virulence, and immune evasion. However, for pathogens that cycle among multiple host species, especially vector-borne pathogens that cycle between disparate species (mammals and arthropods), the impact of genotypic diversity and genotypic interactions on individual genotype transmission and infection success is largely unknown. Vector-borne pathogens, which cause diseases of importance for human and animal health, therefore provide a platform to study how genotypic diversity and interactions among genotypes affect colonization of the vector and resulting pathogen transmission.

Genetic diversity is a hallmark of vector-borne pathogens. Numerous studies have described the circulation and infection of individual hosts or vectors with multiple genotypes of bacterial (e.g., *Anaplasma* sp., *Borrelia* sp.), viral (e.g., West Nile virus, Dengue virus) or protozoal (e.g., *Trypanosoma* sp., *Plasmodium* sp.) vector-borne pathogens [2],[5],[10]–[17]. Competition among vector-borne pathogen genotypes within the mammalian host is common, with competitive success frequently achieved by the more virulent genotype [1],[4],[18]–[22]. For example, in experiments with *P. falciparum* and *B. burgdorferi*, the more virulent genotype replicated to greater levels compared to the competitor, resulting in numerical dominance and preferential transmission. Whether similar genotypic diversity-limiting competition occurs within the arthropod vector is unknown. Further, most studies examine the interactions of only two genotypes at a time; therefore, whether the degree of pathogen genotypic diversity influences the number of genotypes able to infect individual hosts and particularly individual vectors is similarly unknown.

Similar to other tick-borne bacterial pathogens, natural genetic variation within *Francisella tularensis*, including subspecies, is well described [23]–[28]. For example, using multiple loci variable-number tandem repeat analysis on only two loci, 10 unique *F. tularensis* genotypes were recovered from ticks; with the most genotypic diversity found in areas with the greatest prevalence of *F. tularensis* in ticks [23]. The large degree of circulating genotypic diversity observed in that study was indicative of long-standing enzootic transmission of multiple genotypes [23]. Additionally, unlike the majority of tick-borne bacterial pathogens which are refractory to genetic manipulation, *F. tularensis* subsp. *novicida* (herein referred to as *F. novicida*) can be genetically manipulated with relative ease, and thus can serve as a powerful model to address broader questions concerning tick-borne bacterial pathogens. Here, we used a set of differentiable *Francisella novicida* transposon mutants and *Dermacentor andersoni* ticks, which are a natural vector of *Francisella* sp. [29], to investigate how genotypic diversity affects the success of individual genotypes in colonizing the tick vector as compared to the mammalian host. Specifically, we determined (i) if similar numbers of genotypes were able to co-infect mice and ticks, (ii) whether exposure of hosts and vectors to differing numbers of genotypes affected the proportion of genotypes able to be recovered from the host or vector, and (iii) if competition limits the ability of certain genotypes to colonize the vector. To address these questions, pools of *F. novicida* genotypes of varying diversity were inoculated into mice. The genotypes able to infect mice, be acquired by feeding *D. andersoni* nymphs, and persist in the tick midgut through the molt to the adult stage at population and individual host and vector levels were identified. As the tick midgut is the primary site of colonization for most tick-borne pathogens, it serves as a relevant location to examine the effects that varying genotypic diversity has on individual genotype transmission success between host and vector [30],[31]. Finally, we designed a population model to demonstrate how variations in pathogen genotypic diversity, vector and host abundance, and vector-to-host ratios could influence the retention of genotypic diversity in a pathogen population over time.

## Results

### Pathogen diversity is not equally sustained by vector and host populations

We first determined whether the breadth of pathogen genotypic diversity is similarly sustained among mice and ticks at a population level. In all experiments ‘genotypic diversity’ refers to the number of different genotypes, the ‘vector’ refers to the tick and the ‘host’ refers to the mouse. The genotypes ‘available’ to colonize mice and ticks will refer to those genotypes that were inoculated into mice and those genotypes that were detected in terminal mouse blood during peak bacteremia, respectively. Our experiments were initiated by infection of mice, instead of ticks, because of the difficulty and more importantly the variability of artificially infecting ticks.

To simulate diverse genotype populations we used differentiable *F. novicida* transposon-containing genotypes in two large pools (Pool A = 93 genotypes, Pool B = 94 genotypes) each comprised of a different set of *F. novicida* transposon-containing genotypes (Table S1). Genotypes were identified in mouse blood at peak bacteremia (concurrent with completion of nymph feeding) and in adult tick midguts. Ticks fed as nymphs on infected mice over the entire duration of mouse bacteremia and genotypes were identified from the midgut of ticks after the infected nymphs molted to adults. This time point was specifically chosen to avoid detection of genotypes present in the undigested blood meal and confirm that any detected genotype(s) were able to infect and be transstadially maintained in the tick midgut. One limitation of this approach is that we were unable to determine if genotypic diversity was lost prior to or during early infection of the midgut or during transstadial transmission. Our readout of genotype success is colonization of the adult tick midgut, a time point which reflects the cumulative loss of genotypic diversity at any prior point during tick infection. Of the genotypes present in the large-pools, 84% and 81% of Pool A and Pool B genotypes were recovered from their respective mouse cohorts (Table 1). As these large pools encompassed genotypes with variable fitness, it was expected that some genotypes would not be recovered. Of the genotypes that successfully colonized mice, 76% and 54% of genotypes from Pool A and Pool B, respectively, were also acquired by the feeding nymph cohort and transstadially maintained in tick midguts (Table 1). The percentage of genotypes recovered from large-pools was significantly lower for ticks compared to mice (χ^2^ = 13.5, *P* = 0.0002). These results demonstrate that at a population level, despite simultaneous exposure to a large number of genotypes, not all available genotypes colonize mice and ticks. The inability of some *in vitro* generated genotypes to colonize mice was expected given the presence of the introduced transposon; however, the results also suggested additional loss of genotype diversity upon infection of the tick cohort.

**Table 1:** Recovery of *F. novicida* genotypes from populations of mice and ticks exposed to large- or small-pool genotype populations.

	Large genotype pools		Small genotype pools		
	Pool A	Pool B	Pool C	Pool D	Pool E
No. of genotypes in pool	93	94	16	17	16
No. genotypes recovered from mouse cohort (% of total genotypes in pool)	78 (84%)	76 (81%)	16 (100%)	17 (100%)	16 (100%)
No. genotypes recovered from tick cohort* (% of total genotypes recovered from mouse)	59 (76%)	41 (54%)	13 (81%)	15 (88%)	15 (94%)

*all genotypes detected in ticks were also detected in mice.

To determine whether reducing genotypic diversity affected the recovery of genotypes from ticks during mixed-genotype infections, genotypes from pools A and B that had successfully infected mice but were not recovered from ticks were divided into three smaller pools (Pool C = 16 genotypes, Pool D = 17 genotypes, Pool E = 16 genotypes) and the experiment was repeated (Table S1). As expected, all of the genotypes in the small pools (Pools C–E) were recovered from their respective mouse cohorts (Table 1). Interestingly, 81, 88, and 94% of genotypes the from small-genotype pools C, D, and E, respectively, were recovered from their respective tick cohorts despite not being recovered from ticks during the large-genotype pool experiments (Table 1). Similar to the large-pools, the percentage of genotypes recovered from ticks was significantly lower compared to mice (χ^2^ = 6.39, *P* = 0.012). In summary, at a population level, a smaller proportion of available genotypes were recovered from ticks as compared to the mammalian host irrespective of the size of the genotype pool. Further, a greater proportion of available genotypes were recovered from ticks when genotypic diversity was reduced (χ^2^ = 9.30, *P* = 0.0023). These results support that at a population level, *F. novicida* genotype diversity is not equally sustained by mammalian hosts and tick vectors, and suggests that the latter serve as greater ecological filters for *F. novicida* diversity.

### Fewer genotypes are recovered from individual vectors compared to individual hosts

**Figure 1:**
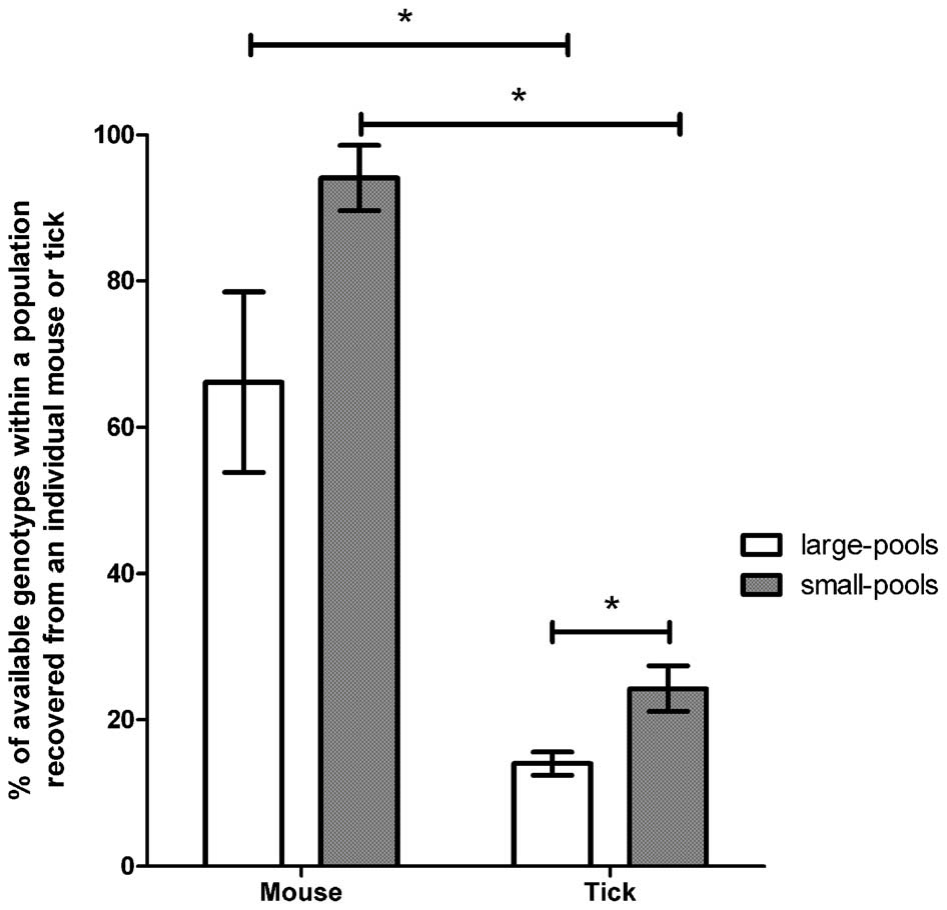
Retention of genotypic diversity in hosts or vectors exposed to large- or small-genotype pools. The mean proportion of genotypes recovered from individual mice or individual ticks were compared between conditions of high and low genotypic diversity. Individual mice exposed to either large- or small-genotype pools were colonized by a significantly greater proportion of genotypes than individual ticks. The proportion of genotypes retained was significantly reduced in ticks exposed to large-genotype pools as compared to ticks exposed to small-genotype pools. Error bars represent ±SEM.

To determine if the observation that the greater reduction in genotypic diversity in the vector population compared to the mammalian host population was also reflected at the level of an individual, we identified the *F. novicida* genotype(s) that colonized individual mice and ticks. For example, if 59 genotypes were recovered from the population of ticks that fed upon mice inoculated with 93 genotypes in Pool A, we determined whether an individual tick was colonized by all or subsets of those 59 genotypes. In the large-genotype pool experiments, individual mice were colonized by a significantly greater percentage of the available genotypes (78 and 53% of the available genotypes in pools A and B, respectively, colonized individual mice) compared with individual ticks (12 and 10% of the available genotypes in pools A and B, respectively, colonized individual ticks) (χ^2^ = 707.4, *P*<0.001) (Figure 1). With regard to ticks in the large-genotype pool experiments, ticks were exposed to a mean of 62 genotypes while feeding on infected mice, and individual ticks were colonized with a mean of 8.5 genotypes (range = 1 to 25, median = 6.5) (Figure S1). These results indicate that the observed genotype diversity sustained by ticks at a population level was the cumulative product of individual ticks infected with subsets of the available genotypes.

To determine if reducing genotypic diversity affected the overall number or proportion of genotypes recovered we identified the genotypes that colonized individual mice and ticks from the small-genotype pool experiments. Similar to the large-genotype pool experiments, a significantly smaller proportion of the available genotypes colonized individual ticks (23, 29, and 21% from Pools C–E, respectively) compared to individual mice (100, 82, and 100% from Pools C–E, respectively) (χ^2^ = 227.5, *P*<0.0001) in the small-genotype pool experiments (Figure 1). In the small-genotype pool experiments overall, ticks were exposed to a mean of 14.3 genotypes and individual ticks were colonized by a mean of 4 genotypes (range = 1 to 11, median = 3.5) (Figure S1).

Examining genotype recovery from individual mice and ticks supported the population level genotype recovery results, and demonstrate that genotype diversity is most severely constrained in the tick. Further, the degree of genotypic diversity influenced both the mean number and proportion of genotypes that colonized ticks. Ticks exposed to more diverse *F. novicida* populations were colonized by a greater total number of genotypes (*Z* = 2.14, *P* = 0.033), but a smaller proportion of the available genotypes (χ^2^ = 44.8, *P*<0.0001) as compared to ticks exposed to less diverse genotype populations (Figure 1, S1).

To determine if the low number of genotypes colonizing ticks compared to mice was the result of a few dominating genotypes, the number of times each genotype was recovered from each tick and mouse was quantified. In general, individual genotypes were recovered from a greater proportion of mice than ticks (Figure S2, S3). On average an individual genotype was recovered from significantly fewer ticks in large pools (11%) compared to small pools (24%) (χ^2^ = 871.1, *P*<0.001). Thus the reduction in genotype diversity during tick infection was not the result of a small subset of genotypes infecting ticks at a greater frequency. Importantly, identification of different genotype combinations from individual ticks that fed upon similarly infected mice indicated that ticks were exposed to a wider array of genotypes then those that were recovered from an individual tick. Further, since ticks fed on mice during their entire duration of bacteremia (approximately 3 days), ticks were likely exposed to all or most or the genotypes identified in the terminal mouse blood. Therefore, the decreased genotype diversity observed in ticks is unlikely to be due to limited sampling opportunities or exposure to a limited number of genotypes.

### Intraspecific competition contributes to reduced genotypic diversity

**Figure 2:**
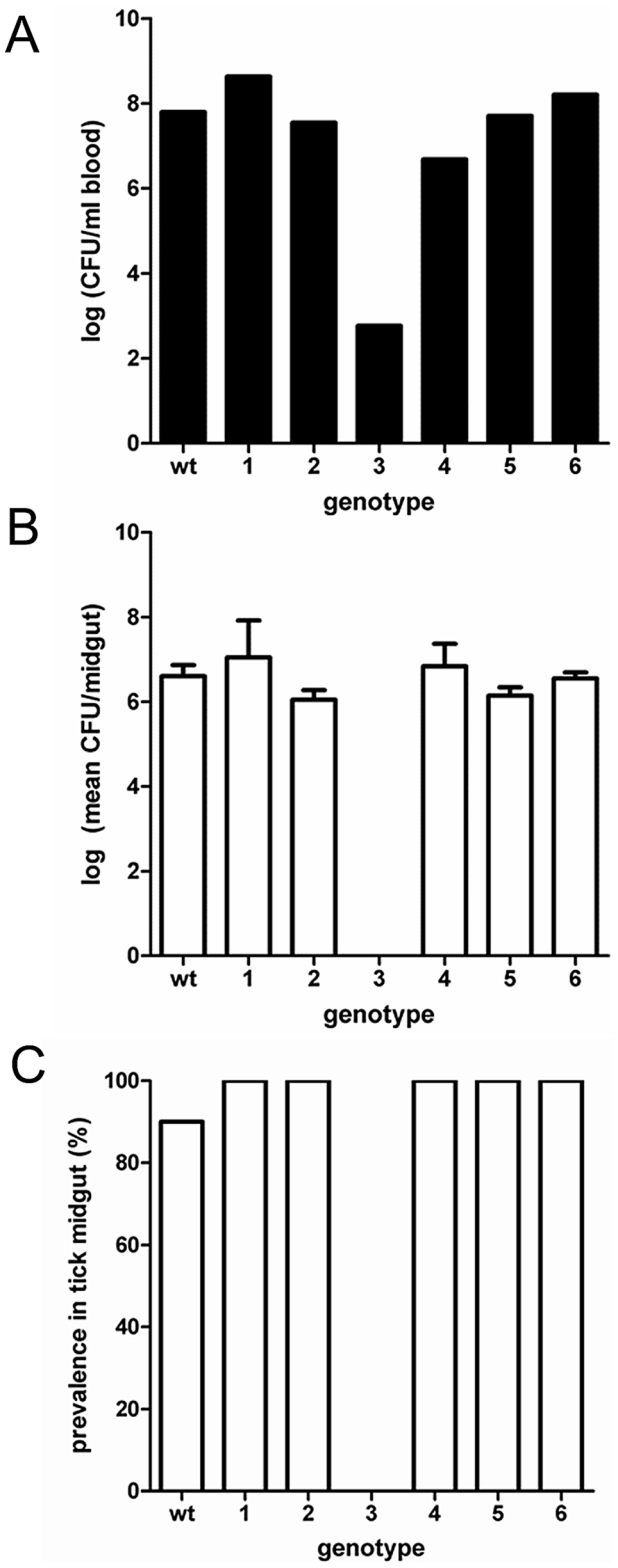
Genotype prevalence and bacterial levels during single-genotype infections. Six genotypes were investigated for their ability to colonize mice and ticks in the absence of other genotypes. A) Bacterial infection level in mouse blood. B) Bacterial infection level in tick midgut. C) Prevalence of genotype colonization in tick midguts. Error bars represent ±SEM.

The reduction in *F. novicida* genotypic diversity upon infection of ticks at both the population and individual level may reflect competition among genotypes. Alternatively, this reduction in diversity may be due to the inability of specific genotypes to infect the tick. To test these hypotheses, the only six genotypes (Genotype 1–6, Table S4) that were consistently recovered from mice but absent from ticks in pooled genotype experiments were further explored. First, we determined if each of these six genotypes, when inoculated individually into mice, were able to colonize feeding ticks. All six genotypes colonized both mice and ticks at infection levels (CFU/ml mouse blood or tick midgut) similar to wild-type with the exception of Genotype 3 that failed to colonize infect ticks (Figure 2A, B) (*F*_5,40_ = 0.88, *P* = 0.50). Moreover, with the exception of Genotypes 3, the other genotypes were recovered from a similar proportion of ticks as wild-type (*P*>0.30 for all comparisons) (Figure 2C).

**Figure 3:**
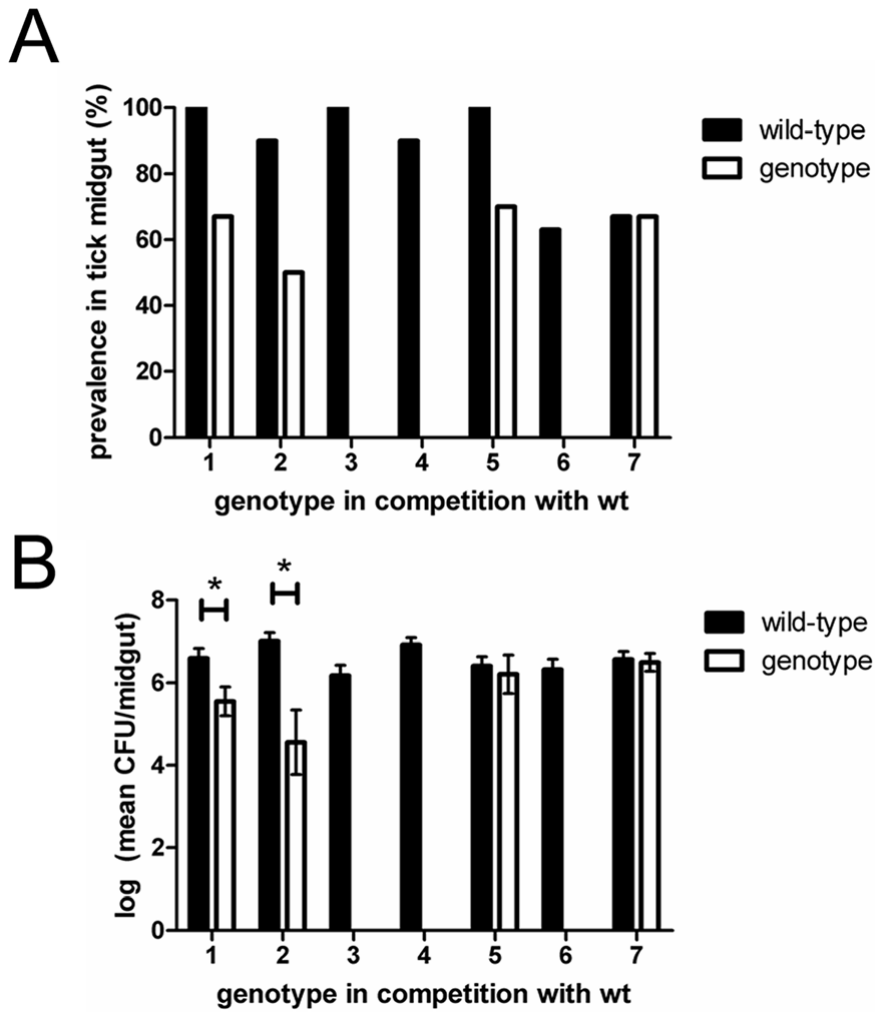
Competition between wild-type and individual genotypes. Competition assays were performed with wild-type and each of the six genotypes not recovered during diverse genotype infections, but recovered from ticks* during individual infection assays. All six genotypes colonized mice during competition with wild-type. A) Genotypes 1, 2, and 5 were recovered from tick midguts with wild-type. B) In co-colonized tick midguts, the mean infection level of Genotype 1 and 2 were significantly lower compared to wild-type. Genotype 7, a genotype similarly fit to wild-type, was used as a positive control to demonstrate that wild-type specifically out-competed Genotypes 1–6. Error bars represent ±SEM. *Genotype 3 was not recovered from ticks in individual infection assays.

As all of these genotypes, except Genotype 3, were competent to infect ticks, each was examined in 1∶1 competition experiments with wild-type to determine if a single additional genotype [wild-type] produced sufficient competition to result in competitive exclusion or suppression of the genotype of interest. In addition to wild-type, the competing genotype in all competition experiments was recovered from the terminal mouse blood (1.1×10^6^, 2.1×10^7^, 2.3×10^3^, 1.9×10^5^, 4.0×10^6^, and 1.6×10^5^ CFU/ml blood for Genotypes 1–6, respectively), thus confirming that ticks were exposed to the genotype of interest during feeding. The mean wild-type bacterial level recovered from terminal blood during competition with individual genotypes was 1.6×10^7^ cfu/ml blood. During competition with wild-type, Genotypes 3, 4 and 6, which had the lowest bacteremia in mice, failed to colonize ticks (Figure 3A). The absence of Genotype 3 in ticks during competition was expected as, when alone, it resulted in a low bacteremia in mice and was not recovered from ticks (Figure 2). The absence of Genotypes 4 and 6 during competition with wild-type is indicative of competitive exclusion as these genotypes, when alone, had similar infection levels in mice and ticks compared to wild-type. When examined individually, both Genotype 4 and 6 were similar to wild-type in terms of both percent infected ticks (χ^2^ = 1.05, *P* = 0.30 for both comparisons) and midgut infection level (*F*_2,26_ = 0.18, *P* = 0.83) (Figure 2B, 2C).

Genotypes 1, 2, and 5 were able to colonize ticks during competition with wild-type (Figure 3); however, a smaller percentage of ticks were colonized by these genotypes compared with wild-type (χ^2^ = 3.60, *P* = 0.058, χ^2^ = 3.81, *P* = 0.051, χ^2^ = 3.53, *P* = 0.060 for genotypes 1, 2, and 5, respectively). In ticks colonized by Genotypes 1, 2, or 5, colonization by wild-type was also observed. Although wild-type could exclude Genotypes 1, 2, or 5 in individual ticks, none of these three genotypes excluded wild-type. Moreover, Genotypes 1 and 2 established significantly lower infection levels in the tick midgut compared to wild-type indicating that these two genotypes were competitively suppressed by wild-type (Figure 3B) for Genotype 1 and wild-type, *t*_13_ = 2.59, *P* = 0.023; for Genotypes 2 and wild-type, (*t*_12_ = 3.87, *P* = 0.0022). Interestingly and despite a lower colonization prevalence compared to wild-type, Genotype 5 achieved infection levels in the tick midgut similar to wild-type (Figure 3) (*t*_15_ = 0.42, *P* = 0.68).

As a control to demonstrate that wild-type specifically out-competed Genotypes 1–6, we performed a 1∶1 competition assay with wild-type and Genotype 7, which has a transposon in a non-coding region (isftu-2), and behaves similarly to wild-type in both mice and ticks [29]. In the terminal mouse blood the bacterial levels for wild-type and Genotype 7 were 9.3×10^6^ and 7.0×10^6^ CFU/ml blood, respectively, confirming ticks were exposed to both genotypes. Equal proportions of ticks were colonized by Genotype 7 and wild-type together, Genotype 7 alone, and wild-type alone. In ticks that were co-infected, both Genotype 7 and wild-type achieved similar infection levels in the tick midgut (Figure 3) (*t*_6_ = 0.25, *P* = 0.81). The equal success of Genotype 7 and wild-type in colonizing ticks during competition with one another demonstrated that Genotypes 1–6 were diminished or excluded due to competition rather than random effects. In summary, these results indicate that Genotypes 1–6 have a fitness disadvantage in the vector as compared to wild-type as co-infection of any of these genotypes with wild-type results in their competitive exclusion (e.g., Genotype 3, 4, and 6) or competitive suppression (e.g., Genotypes 1, 2, and 5). This demonstrates that co-infection with a single, more fit genotype is sufficient to alter the success of the competing genotype even if the less fit competitor is competent upon single-infection. Further, both competitive suppression and competitive exclusion offer explanations for the loss of genotypic diversity observed during pathogen infection of ticks.

### Modeling effects of hosts and vectors on pathogen diversity

Our experiments suggest that pathogen genotypic diversity is restricted within the tick vector at both population and individual levels. This restriction in diversity is most pronounced within individual ticks, suggesting that the abundance of ticks will strongly affect pathogen genotypic diversity within an environment. To further explore how variations in vector and host populations influence pathogen genotypic diversity, we developed a simple population model that incorporated data from our experiments. The model contained separate functions for vectors (ticks), hosts (mice), and pathogens (*Francisella* genotypes) (Figure S4). We used this model to investigate how vector-to-host ratios, vector and host abundance, and the initial number of pathogen genotypes within a population influenced the overall maintenance of genotype diversity in the population.

**Figure 4:**
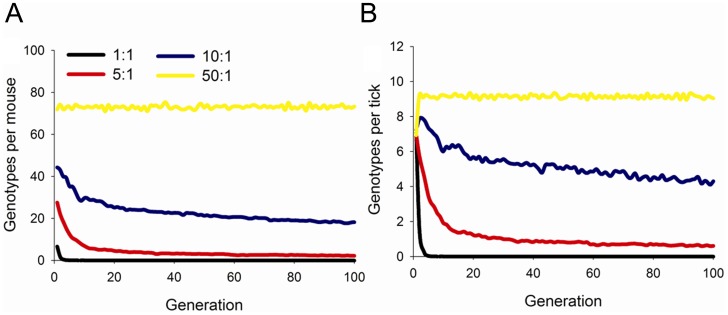
Maintenance of genotypic diversity in mice and ticks over 100 generations. Number of pathogen genotypes per (A) mouse and (B) tick in model simulations with 100 mice and varying vector-to-host ratios. Variation in vector-to-host ratios was simulated by varying the number of ticks from 100 (1∶1 ratio) to 5000 (50∶1 ratio).

With all model conditions, individual mice harbored greater pathogen genotypic diversity than ticks (Figure 4, S5, S6). Thus, rare pathogen genotypes were more likely to be lost from the vector population than from the mammalian host population. At the population level, vector-to-host ratios strongly influenced the retention of pathogen genotypic diversity (Figure 4). When vector densities declined and vector-to-host ratios approached 1, pathogen genotypic diversity rapidly declined as individual genotypes were lost from the system. In contrast, high vector-to-host ratios increased the retention of genotypic diversity because the filtering effects of individual ticks were reduced due to large population sizes (Figure 4). Variation in vector or host abundance did not influence pathogen genotypic diversity as strongly as vector-to-host ratios; however, in general, larger vector and host populations led to greater maintenance of pathogen genotypic diversity (Figure S5). Initial pathogen genotypic diversity also influenced the number of pathogen genotypes maintained in the vector and host populations (Figure S6). Not surprisingly, both vectors and hosts individually harbored more pathogen genotypes when the number of initial genotypes was greater. However, the proportion of genotypes in the population infecting individual vectors and hosts declined with greater initial pathogen genotypic diversity (Figure S6) as observed in our experiments with large- and small-genotype pools (Figure 2). Thus, the model showed a trade-off between the raw number of pathogen genotypes that infected individual vectors and hosts and the proportion of the pathogen genotype population they represented.

## Discussion

Our results demonstrate that pathogen genotypic diversity is restricted to a greater degree in the tick vector as compared to the mammalian host. Moreover, the extent to which hosts and vectors contribute to the maintenance of pathogen genotypic diversity is influenced by the initial degree of genotypic diversity in the pathogen population and competition among genotypes during infection of the vector. Within a host or vector, competitive interactions among genotypes can result in the reduction or elimination of one or more genotypes. Studies on co-infecting *Plasmodium* genotypes illustrate how a more virulent genotype can competitively suppress or prevent a less virulent genotype from being transmitted between the mammalian host and mosquito vector [32]–[35]. Additionally, studies on arboviruses such as West Nile virus and Dengue virus have demonstrated that genotypic diversity can be correlated with transmissibility or virulence [7],[9]. Similar to our results, intrahost examination of West Nile virus revealed that viral genetic diversity was restricted in mosquito midguts compared to the input pool [36],[37]. Interestingly, however, despite a reduction of viral diversity in the mosquito midgut, corresponding salivary samples were similar in diversity to the input pool, perhaps contributed to accumulation of mutations as a result of relaxed purifying selection during infection of the mosquito [36].

In this study, *F. novicida* genotype diversity was not equally sustained by mice and ticks, and the greatest restriction in genotypic diversity occurred in individual ticks. This reduction in diversity was mediated by a combination of both stochastic and selective forces, and was unlikely to be an artifact of tick feeding. Based on our results, despite exposure to a large array of mutants, individual ticks were not able to support the same number of mutants as mice. One possible reason for genotypic restriction is that resources for bacterial colonization, such as nutrient availability or receptors for cell entry, are more limited in ticks than in mice, which could lead to competition among genotypes for limited resources. Several lines of evidence suggest that strong competition among genotypes occurred in ticks. First, individual ticks that fed upon the same mouse infected with up to 94 genotypes were colonized by different combinations of genotypes. Second, five genotypes not recovered from ticks during pooled-genotype experiments were competent to colonize ticks, in most cases to wild-type levels, in the absence of a second genotype. Third, in competition assays with wild-type and a wild-type-like genotype (Genotype 7), both were equally able to compete and colonize ticks, which further indicated that the absence of Genotypes 1–6 from the pooled-genotype experiments was not random. Our experimental design allowed us to examine the genotypic diversity that was sustained by ticks from the genotypes present during the nymphal blood meals to recovery of genotypes from adult tick midguts. This period of time encompassed several points where genotypic diversity could have been lost in the tick midgut including during initial entry into the nymph midgut, early replication and colonization events in the nymph midgut, transstadial transmission from nymph to adult, or continued colonization in the adult midgut. Although our results clearly demonstrate that competition is occurring among *F. novicida* genotypes during infection of the tick vector, it is interesting to note that a previous study speculated that facilitative interactions among genotypes in mixed *B. burgdorferi* genotype infections conferred an advantage for the bacteria to establish and maintain infection in ticks [15],[38]. It is possible that such interactions may occur in this system.

Additional variables that could further influence competition among genotypes and contribute to the observed reduction in genotype diversity in ticks include the infection level for an individual genotype, transmission priority (the order in which genotypes are transmitted), and genotype fitness. With regard to the latter two variables, our results indicated that reduction in fitness can in some instances overwhelm the stochastic forces that dictate tick infection by pathogen genotypes [29],[39],[40]. Overall, *F. novicida* bacterial levels did not vary based on genotype diversity and were similar to previously reported single-genotype infection levels [29]. This suggests that ticks have an infection threshold limit for *F. novicida*, such that as the number of genotypes a tick is exposed to increases, the maximum infection level of any individual genotype is proportionately reduced [29]. Therefore, genotypes that are able to replicate first will have a greater opportunity to colonize the tick while reducing the amount of available resources for incoming genotypes (founder effect) [12]. Additionally, greater numerical success in one host or vector will confer a greater probability of subsequent transmission.

The transmission priority of genotypes between mice and ticks was stochastic, such that ticks had an opportunity to acquire the genotypes that colonized mice relative to the genotype-specific infection level in mice. Transmission priority is potentially important if resources are more limited within the tick and monopolized by genotypes on a “first come, first serve” basis. In most pooled-genotype experiments, genotype recovery was random and ticks were colonized by small subsets of the available genotypes in different combinations. Although we strived to initiate our pooled-genotype experiments with equal ratios of genotypes, four genotypes in Pool B were recovered from a greater percentage of mice and ticks implying that they had a numerical advantage in the initial inoculum, maintained that advantage while colonizing mice and were available at a greater frequency for feeding ticks to acquire (Figure S2). These four genotypes, which were recovered from a greater percentage of ticks than the other genotypes comprising Pool B, provide evidence for genotypes with an initial advantage having greater transmission and colonization success. Importantly, although these four genotypes were identified in a greater percentage of ticks, they were not the sole genotypes observed and were commonly identified in individual ticks with less frequently occurring genotypes. These results are similar to those of a *Trypanosoma brucei* study and recently a *B. burgdorferi* study where vector acquisition of genotypes from mice, infected with multiple, similarly fit pathogen genotypes, was noted as random and the first genotype able to infect an individual vector had an advantage during dissemination to other tissues and in subsequent transmission [12],[41]. Stochastic forces also play a prominent role in shaping arboviral transmission, and has been demonstrated for West Nile Virus and Venezuelan equine encephalitis virus [42],[43].

Genotype fitness can influence competitive ability as well as virulence as demonstrated by co-infection studies using genotypes with known fitness differences [33]–[35]. A range of fitness among the *F. novicida* genotypes examined was expected, depending on the location of the transposon. The overall genetic similarly of genotype populations suggests that the majority likely shared similar abilities to infect mice and ticks (Table 1). We surmised that the six genotypes absent from ticks in the pooled-genotype experiments were out-competed. This postulation was supported by the results of the 1∶1 competition assays between wild-type and Genotypes 1–6, where wild-type succeeded disproportionately in terms of infection prevalence and infection load compared to the competing genotype. The competition assay between wild-type and Genotype 7 confirmed that if Genotypes 1–6 had been similarly fit as wild-type, they would have succeeded to a similar extent as Genotype 7 did during competition with wild-type. The finding that Genotypes 1–6 were able to colonize ticks during single-genotype experiments but not in during competition with more fit genotypes supported the notion that the location of the transposon in these genotypes exacts some fitness cost, although the exact mechanism by which this is occurring remains unknown.

In the field, genotypic diversity is likely to be dynamic and heavily influenced by environmental variables. Genotypic diversity, when measured, generally occurs as insertions, deletions, and polymorphisms in individual and small numbers of nucleotides [44]–[46]. Additionally, gene duplications and deletions do occur [47]. While insertions are over-represented in our population, the use of naturally occurring genotypes is not possible, as a collection of greater than 150 different genotypes that can easily be distinguished one from another do not exist for any tick borne bacterial pathogen. Importantly, the alterations in phenotype in our population are likely highly variable and represent a broad spectrum, from complete knock-out of gene function to no alteration in gene function. Thus, while the type of genetic mutation represented in our population is limited as compared to a natural population, a broad spectrum of alterations in phenotype is likely to be represented. Further, in our experiments more mutational robustness was observed in the vertebrate, however, within a host infected with naturally occurring genotypes those genotypes could possess very different fitness abilities, thus altering the outcome of within-host interactions and ongoing transmission.

To extrapolate our results to a broader range of field scenarios we created a model to explore how variations in vector-to-host ratio, vector and host abundance, and initial pathogen genotypic diversity affected the retention of pathogen genotypic diversity in a population over time. These variables were selected because our experimental data indicated that the greatest restriction in *F. novicida* genotype diversity occurred during colonization of ticks compared to mice. We assumed that there was no mortality of vectors and hosts, and thus the model likely over-estimated the conservation of diversity (as pathogen genotypes might be lost from dying vectors and hosts). Our modeling results suggested that local extinction of pathogen genotypes, and genotypic diversity overall, is more likely to be affected during pathogen infection of ticks. Vector-to-host ratio was the most important variable in the maintenance of pathogen genotypic diversity over time in a population; however, abundance of vectors and hosts, and initial pathogen genotypic diversity also contributed. Finally, our model was conservative in design in that it assumed equal fitness among genotypes, that all ticks fed, and does not incorporate the addition of new genotypes beyond those initially present. If additional values are known, derivations of this model could be used to examine these variables which could result in accelerated specific genotype extinction or retention. Although our model featured a tick-borne pathogen, our experimental results and model predictions are in line with epidemiological data of other vector-borne pathogens, including *Plasmodium* spp., where areas of high transmission are associated with abundant vector populations that collectively support a great diversity of pathogen genotypes [6].

Most vector-borne pathogen studies examining genotype co-infection to date either survey the circulating pathogen genotypes in an area or conduct competition assays among pairs of genotypes, frequently differing drastically in fitness (e.g., attenuated versus virulent, transmissible versus not transmissible) [2],[5],[11],[15],[19],[48]–[50]. Knowledge gaps exist regarding the role of the vector in supporting or restricting pathogen genotype diversity in a population. In this study, both the experimental data and population modeling data revealed that the tick vector acted as a greater ecological filter for pathogen genotypic diversity compared to the mammalian host. This restriction of *F. novicida* genotypic diversity in ticks was further affected by the initial amount of genotypic diversity and competition among genotypes. Extrapolation of our results in a model revealed variables, including vector-to-host ratio, which over many generations played important roles in the maintenance of pathogen genotypic diversity.

The marked reduction in genotypic diversity within the tick indicates that intervention strategies targeting the pathogen within the tick, such as introduction of highly competitive genotypes, are likely to be effective in disrupting disease transmission. Further, understanding how pathogen genotypic diversity and genotype interactions within the host and vector affect colonization success is essential to understanding pathogen transmission, selection and disease ecology.

## Materials and Methods

### Ethics statement

This study was carried out in accordance with the following: Animal Welfare Act (9 CFR Ch. 1 Subpart C 2.31 (c) (1–8)), Guide for the care and use of Agricultural Animals in Agricultural Research and Training (Chap.1), and the Public Health Service Policy on Humane Care and Use of Laboratory Animals (Section IV.B. (1–8)). All protocols involving the use of animals were approved by the Washington State University Institutional Animal Care and Use Committee (IACUC) (ASAF Number: 3686 and 4430).

### Ticks, mice and rabbits

*Dermacentor andersoni* (Reynold's Creek) nymphs were obtained from a colony maintained by USDA-ARS-ADRU (Pullman, WA). All nymphs were fed on C57BL/6 mice [29]. After inoculation with *F. novicida*, mice were monitored twice daily for signs of illness. At the onset of severe illness (ruffled fur, hunched posture, ocular or nasal discharge, ataxia, etc), mice were euthanized and blood was cultured to determine bacteremia as described in the following section. If mice did not develop disease, they were euthanized upon completion of nymph feeding and bacteremia similarly determined via culture. In some experiments, adult *D. andersoni* were fed to repletion on male New Zealand white rabbits (Western Oregon Rabbit Company, Philomath, OR) [29]. Rabbits remained asymptomatic and were culture negative at the end of tick feeding.

### Bacterial culture conditions

Wild-type *F. novicida* (U112) or transposon mutants containing a kanamycin resistance cassette [51] (Table S1) were used in all experiments. All *F. novicida* mutant genotypes were cultured in tryptic soy broth (TSB) or on tryptic soy agar (TSA) containing 0.1% L-cysteine and kanamycin (15 µg/ml) (kanamycin was omitted when culturing wild-type *F. novicida*) [29]. Briefly, *F. novicida* broth cultures were incubated at 37°C and 225 rpm either overnight or for 3 hr, depending on the experiment. *F. novicida* agar cultures were incubated at 37°C for 48 hrs and the resulting colony forming units (CFU) enumerated. To recover *F. novicida* from blood, whole blood was plated from individual mice. To recover *F. novicida* from ticks, tick midguts were individually dissected and homogenized in Lysing Matrix H tubes (MP Biomedical, Solon, OH) containing 500 µl of 1× PBS for 13 seconds at 3 M/s and plated.

### Discrimination of *F. novicida* genotypes

For all samples, genomic DNA (gDNA) was isolated from lawns (>10,000 CFU) of *F. novicida* culture using a DNeasy kit (Qiagen, Valencia, CA). Individual *F. novicida* genotypes were identified by PCR amplification of a 350–700 bp fragment using a universal primer located within the kanamycin cassette and a genotype-specific primer in the adjacent sequence (Table S2). Individual reactions included 2× GoTaq Mastermix (Promega, Madison, WI), 2.5-µM of each primer, and 50-ng of gDNA template. Thermocycler conditions were as follows: Step 1 (×1), 94°C for 2 min; Step 2 (×35), 94°C for 45 sec, 54°C for 45 sec, 72°C for 45 sec; Step 3 (×1), 72°C for 5 min. Following electrophoresis, PCR products were visualized on a 2% agarose gel containing SYBR Safe (Invitrogen, Carlsbad, CA).

### Diverse genotype infection assays

To simulate diverse genotype infections, clones from two randomly chosen plates (NR-8058 and NR-8065, BEI Resources, Manassas, VA) from a *F. novicida* transposon mutant library were used to assemble the large pools (Pool A, n = 93; B, n = 94) for infection assays (Table S1). The populations of genotypes that comprised the small pools (Pool C, n = 16; D, n = 17; E, n = 16) (Table S1), were those that were recovered from the mouse blood but not the tick midgut in the large pool infection assays.

To generate diverse inocula, glycerol stocks of individual *F. novicida* genotypes were each inoculated into a single well in a 96-well plate containing 1 ml of TSB and grown overnight. Overnight cultures were sub-inoculated into fresh TSB for a starting concentration of 1∶1500 (1 µl overnight culture into 1.5 ml of TSB). Cultures were incubated for 3 hr and 50 µl of individual genotype cultures were combined to generate the mixed genotype inocula. An OD600 measurement was obtained for the combined culture and the appropriate dilutions were made in 1× PBS for a final concentration of 4000 CFU (∼40 CFU/genotype) or 1000 CFU (∼60 CFU/genotype) in 100 µl for the large- and small-pool infection assays, respectively.

Mice infested with *D. andersoni* nymphs were intraperitoneally inoculated (6 mice/pool and 3 mice/pool for the large and small pool infection assays, respectively). To verify that all genotypes were present in the inoculum, an aliquot was plated, allowed to grow to a lawn (>10,000 CFU) and re-suspended in 5 ml 1× PBS, from which 100 µl was used for gDNA extraction, and examined by genotype-specific PCR. Terminal mouse blood was plated and the resulting bacterial cultures examined to determine the bacterial load and identify the genotypes that successfully infected the mice and thus were available for the feeding nymphs to acquire. After feeding, nymphs were incubated at 25°C and allowed to molt to adults. For the large pool infection assays, the infected adult ticks were fed on a naïve rabbit to expand the *F. novicida* infection load in the tick midgut; however, we later determined this extra feeding was not necessary to detect the population of genotypes in the tick midgut and was omitted in subsequent infection assays. Once molting to adults was complete, midguts were dissected from individual ticks, homogenized and plated, and the resulting bacterial lawns were examined to determine the bacterial level and identify the genotypes that had colonized the tick midgut and had been transstadially maintained. Bacterial lawns derived from blood (n = 3) or midgut cultures (n = 10 to 12) were processed as described above from individual mice or ticks and the *F. novicida* genotype population determined from individual or pooled (combine aliquots of re-suspended culture from like inoculated/exposed mice or ticks) samples.

### Individual and competition infection assays

In the infection assays using multiple genotypes, described above, six transposon-containing genotypes were consistently recovered from the mouse blood but not the tick midgut. These genotypes were then tested in individual infection assays and competition assays with wild-type. As a control for the competition assays, a transposon-containing genotype that has a phenotype similar to that of wild-type [29] was used. For individual genotype infection assays, the inoculum was prepared as previously described with each mouse receiving 1000 CFU of a single genotype. Detection of the individual genotype in the inoculum, mouse blood, and tick midgut was accomplished by culture and the identity of the genotype was verified by PCR.

For competition assays, a 1∶1 ratio (500∶500 CFU) of two different genotypes were injected into mice in the same manner as described above. To enumerate each genotype within blood or midgut, CFUs were calculated by dual plating samples on antibiotic-free and kanamycin-containing TSA plates. This allowed enumeration of the transposon-containing genotype (CFU on kanamycin-containing TSA plates) and wild-type (CFU on antibiotic-free plates minus CFU enumerated on the reciprocal kanamycin-containing TSA plates). The ratio of wild-type to transposon-containing genotype was determined for each competition assay in the inoculum, mouse blood, and adult tick midgut. Six to twelve ticks were assessed for each competition pairing.

### Diversity model

We developed a population model that incorporated data from the experiments to explore how variation in vector and host populations would influence pathogen genotypic diversity over a range of vector and host conditions. The model contained functions for vectors (ticks), hosts (mice), and pathogens (*Francisella* genotypes) (Figure S5). The model was initiated by allocating pathogen genotypes to a population of mice, with each mouse receiving all pathogen genotypes. These pathogen genotypes were then tracked over time in both tick and mice populations. The model had a generational time step, and at each time step uninfected ticks attached to mice, fed, and acquired pathogens. Infected ticks then molted and fed on uninfected mice (i.e., the next generation), transmitting pathogens in the process (Figure S5).

The model was individual-based, such that each tick only acquired pathogens from the mouse it fed on; similarly, mice only acquired pathogens from ticks that fed upon them. The probability that an uninfected tick acquired pathogen genotype *g* from mouse *m* 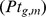 was:
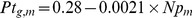
(1)where 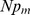 is the number of pathogen genotypes harbored by mouse *m*. Thus, the maximum probability of a tick acquiring any genotype was 28%. The model was stochastic, and a random number was drawn from a uniform distribution between 0–1 and compared with 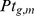 to determine whether ticks acquired each pathogen genotype. In turn, the probability that an uninfected mouse acquired a pathogen genotype *g* from ticks was as follows 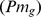:
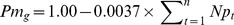
(2)where 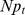 is the number of pathogen genotypes harbored by tick *t*. The summation adds up the total number of genotypes for all ticks that fed on each particular mouse (total = *n*). Thus, the maximum probability of a mouse acquiring any genotype was 100%. Like ticks, mice were modeled individually and the pathogen genotypes they acquired were stochastic. Equations for *Pt* and *Pm* were generated by fitting linear model to data from the experiments.

One limitation of the model is that using linear functions sets a maximum acquisition value that may be lower than the probability for “fit” genotypes. However, such functions were used to approximate the average genotype. This was done because it is difficult to assume the proportion of genotypes that would be “fit” (i.e., have a higher acquisition probability) and “unfit” (i.e., have a lower acquisition probability) in natural populations; therefore, we only modeled the “average genotype”. We did explore alternative forms of the acquisition function with a greater maximum acquisition value. However, with any form of the model our qualitative results on the role of vector-to-host density, initial vector and host abundance, and initial pathogen diversity did not change. Thus, we only present results of this simple model that did not distinguish between genotypes in terms of fitness. While simple, results with this model were used to demonstrate how diversity might be maintained in natural population with varying conditions.

In the baseline set of simulations, there were 100 mice, 1,000 ticks and 100 pathogen genotypes. However, these values were varied in sensitivity analyses to investigate the effects of different vector-to-host ratios, differences in vector and host abundance, and different initial pathogen genotypic diversity on the maintenance of pathogen genotypic diversity over time (Table S3). For each set of initial conditions, the model was run for 100 generations to examine the maintenance of pathogen genotypic diversity over time in ticks and mice. For each set of initial model conditions (Table S3), we ran the model 1,000 times to account for the stochastic nature of the model. Results presented represent the average values from these 1,000 simulations.

### Statistical analysis

All statistical analyses were conducted using JMP Statistical Discovery Software Version 11 (Cary, NC). We used logistic regression to explore effects of genotype diversity (i.e., pool size), genotype group nested within pool size, and host (mouse vs. tick), and all two-way interactions on the recovery of genotypes from hosts and vectors. Genotype group was not significant in these analyses, (χ^2^ = 3.36, *P* = 0.34), and so final analyses were only run with the factors genotype diversity, host, and their interaction. In these analyses, the number of genotypes recovered or not recovered from hosts and vectors were binomial count data. To look at the number of genotypes recovered from individual ticks in differing pool sizes (large vs small) we used non-parametric Wilcoxon tests, as data on the number of genotypes recovered were non-normal. For single wild-type or genotype infection assays, we used an ANOVA to compare *F. novicida* genotype bacterial levels to wild-type bacterial levels during single-genotype infection experiments. For 1∶1 competition experiments between wild-type and a select genotype, we used two-sample *t*-tests to compare wild-type and genotype bacterial levels in tick midguts. Moreover, we used Fisher's exact tests to determine the proportion of ticks that were infected with each genotype compared to wild-type in these 1∶1 competition assays. For all analyzes, an α value of 0.05 was used to determine statistical significance.

## Supporting Information

Figure S1 Number and proportional recovery of genotypes from ticks exposed to genotype populations varying in diversity. Comparison of the mean genotype recovery in individual ticks that fed upon mice inoculated with large- or small- genotype pools as the (A) number of genotypes recovered and the (B) proportion of available genotypes recovered. A significantly greater number of genotypes were recovered in large-genotype pools compared to small-genotype pools (*t* = 3.783, *P* = 0.0006); however, a significantly greater proportion of the total available genotypes were recovered in small-genotype pools compared to large-genotype pools (*t* = 3.011, *P* = 0.0044).
(TIF)

Figure S2 Recovery of individual genotypes from mice and ticks exposed to large-genotype pools. Frequency of individual genotype recovery from (A) mice and (B) ticks exposed to Pool A genotypes. Frequency of individual genotype recovery from (C) mice and (D) ticks exposed to Pool B genotypes. The value included above each graph and the red dashed line indicates the mean number of times a single genotype was recovered from mice and ticks. A significantly higher proportion of genotypes were recovered from mice compared with ticks (χ^2^ = 501.8, *P*<0.0001).
(TIF)

Figure S3 Recovery of individual genotypes from mice and ticks exposed to small-genotype pools. Frequency of individual genotype recovery from mice, (A) Pool C, (B) Pool D, (C) Pool E. Frequency of individual genotype recovery from ticks, (D) Pool C, (E) Pool D, (F) Pool E. The value above each graph and the red dashed line indicates the mean number of times a single genotype was recovered from mice and ticks. A significantly higher proportion of genotypes were recovered from mice compared with ticks (χ^2^ = 267.0, *P*<0.0001).
(TIF)

Figure S4 Flowchart of the population model. Shown are the first two generations of the model. Lines represent feeding events and/or pathogen transfer events. To initiate the model, all pathogen genotypes are allocated to all mice. These mice are fed upon by ticks, with all ticks finding a host (not all mice are fed upon by the same number of ticks), and pathogen genotypes are acquire by feeding ticks. Infected ticks then feed on naïve mice in the next generation, mixing the tick population and resulting in transmission. These mice are then fed upon by second generation naïve ticks. The cycle continues for a specified number of generations.
(TIF)

Figure S5 Retention of genotype diversity as a function of varying host and tick abundance. Number of pathogen genotypes per (A) mouse and (B) tick in simulations with varying abundances of mice and ticks. In all simulations the vector-to-host ratio was 10∶1, but the number of vectors and hosts was varied.
(TIF)

Figure S6 Retention of genotype diversity in individual mice and ticks as a function of the number of genotypes in a population. Number of pathogen genotypes per (A) mouse and (B) tick, and the proportion of pathogen genotypes per (C) mouse and (D) tick, in simulations with varying initial number of pathogen genotypes. In all simulations the vector-to-host ratio was 10∶1 with 100 mice and 1000 ticks, but the number of initial pathogen genotypes was varied.
(TIF)

Table S1 *F. novicida* transposon mutants used in this study.
(PDF)

Table S2 List of primers used in this study.
(PDF)

Table S3 Parameters used in the standard runs of the model for vectors, hosts, and pathogens, and values used in the sensitivity analyses of vector-to-host ratios, vector and host abundance, and initial pathogen genotypic diversity.
(DOCX)

Table S4 List of genotypes, recovered from mice but not ticks in pooled genotype experiments, that were further investigated in single-infection assays and 1∶1 competition assays with wild-type.
(DOCX)

## References

[1] Read AF, Taylor LH (2001) The ecology of genetically diverse infections. Science 292: 1099–1102. 11352063 10.1126/science.1059410

[2] Ladbury GA, Stuen S, Thomas R, Bown KJ, Woldehiwet Z, et al (2008) Dynamic transmission of numerous *Anaplasma phagocytophilum* genotypes among lambs in an infected sheep flock in an area of anaplasmosis endemicity. Journal of Clinical Microbiology 46: 1686–1691. 18367562 10.1128/JCM.02068-07 PMC2395098

[3] Mideo N (2009) Parasite adaptations to within-host competition. Trends in Parasitology 25: 261–268. 19409846 10.1016/j.pt.2009.03.001

[4] Balmer O, Tanner M (2011) Prevalence and implications of multiple-strain infections. The Lancet Infectious Diseases 11: 868–878. 22035615 10.1016/S1473-3099(11)70241-9

[5] Tsao K, Bent SJ, Fish D (2013) Identification of *Borrelia burgdorferi ospC* genotypes in host tissue and feeding ticks by terminal restriction fragment length polymorphisms. Applied and Environmental Microbiology 79: 958–964. 23183976 10.1128/AEM.03106-12 PMC3568573

[6] Anderson TJ, Haubold B, Williams JT, Estrada-Franco JG, Richardson L, et al (2000) Microsatellite markers reveal a spectrum of population structures in the malaria parasite *Plasmodium falciparum* . Molecular Biology and Evolution 17: 1467–1482. 11018154 10.1093/oxfordjournals.molbev.a026247

[7] Cologna R, Armstrong PM, Rico-Hesse R (2005) Selection for virulent dengue viruses occurs in humans and mosquitoes. J Virol 79: 853–859. 15613313 10.1128/JVI.79.2.853-859.2005 PMC538581

[8] Smith T, Beck HP, Kitua A, Mwankusye S, Felger I, et al (1999) Age dependence of the multiplicity of *Plasmodium falciparum* infections and of other malariological indices in an area of high endemicity. Transactions of the Royal Society of Tropical Medicine and Hygiene 93 Suppl 1: 15–20. 10.1016/s0035-9203(99)90322-x 10450421

[9] Ebel GD, Carricaburu J, Young D, Bernard KA, Kramer LD (2004) Genetic and phenotypic variation of West Nile virus in New York, 2000–2003. Am J Trop Med Hyg 71: 493–500. 15516648

[10] Day KP, Koella JC, Nee S, Gupta S, Read AF (1992) Population genetics and dynamics of *Plasmodium falciparum*: an ecological view. Parasitology 104 Suppl: S35–52. 1589299 10.1017/s0031182000075235

[11] Swanson KI, Norris DE (2008) Presence of multiple variants of *Borrelia burgdorferi* in the natural reservoir *Peromyscus leucopus* throughout a transmission season. Vector Borne and Zoonotic Diseases 8: 397–405. 18399776 10.1089/vbz.2007.0222 PMC2978052

[12] Oberle M, Balmer O, Brun R, Roditi I (2010) Bottlenecks and the maintenance of minor genotypes during the life cycle of *Trypanosoma brucei* . PLoS Pathogens 6: e1001023. 20686656 10.1371/journal.ppat.1001023 PMC2912391

[13] Manske M, Miotto O, Campino S, Auburn S, Almagro-Garcia J, et al (2012) Analysis of *Plasmodium falciparum* diversity in natural infections by deep sequencing. Nature 487: 375–379. 22722859 10.1038/nature11174 PMC3738909

[14] Romano CM, Lauck M, Salvador FS, Lima CR, Villas-Boas LS, et al (2013) Inter- and intra-host viral diversity in a large seasonal DENV2 outbreak. PloS One 8: e70318. 23936406 10.1371/journal.pone.0070318 PMC3732279

[15] Andersson M, Scherman K, Raberg L (2013) Multiple-strain infections of *Borrelia afzelii*: a role for within-host interactions in the maintenance of antigenic diversity? The American Naturalist 181: 545–554. 10.1086/669905 23535618

[16] Mann BR, McMullen AR, Guzman H, Tesh RB, Barrett AD (2013) Dynamic transmission of West Nile virus across the United States-Mexican border. Virology 436: 75–80. 23141421 10.1016/j.virol.2012.10.023 PMC4142591

[17] Rudenko N, Golovchenko M, Belfiore NM, Grubhoffer L, Oliver JH Jr (2014) Divergence of *Borrelia burgdorferi* sensu lato spirochetes could be driven by the host: diversity of *Borrelia* strains isolated from ticks feeding on a single bird. Parasites & Vectors 7: 4. 24383476 10.1186/1756-3305-7-4 PMC3892016

[18] Anderson RM (1999) The pandemic of antibiotic resistance. Nature Medicine 5: 147–149. 10.1038/5507 9930857

[19] Policastro PF, Raffel SJ, Schwan TG (2013) *Borrelia hermsii* acquisition order in superinfected ticks determines transmission efficiency. Infection and Immunity 81: 2899–2908. 23716615 10.1128/IAI.00542-13 PMC3719565

[20] Wargo AR, de Roode JC, Huijben S, Drew DR, Read AF (2007) Transmission stage investment of malaria parasites in response to in-host competition. Proceedings Biological Sciences/The Royal Society 274: 2629–2638. 10.1098/rspb.2007.0873 PMC1975767 17711832

[21] Bell AS, de Roode JC, Sim D, Read AF (2006) Within-host competition in genetically diverse malaria infections: parasite virulence and competitive success. Evolution; International Journal of Organic Evolution 60: 1358–1371. 16929653

[22] Troy EB, Lin T, Gao L, Lazinski DW, Camilli A, et al (2013) Understanding barriers to *Borrelia burgdorferi* dissemination during infection using massively parallel sequencing. Infection and Immunity 81: 2347–2357. 23608706 10.1128/IAI.00266-13 PMC3697624

[23] Goethert HK, Shani I, Telford SR 3rd (2004) Genotypic diversity of *Francisella tularensis* infecting *Dermacentor variabilis* ticks on Martha's Vineyard, Massachusetts. Journal of Clinical Microbiology 42: 4968–4973. 15528681 10.1128/JCM.42.11.4968-4973.2004 PMC525218

[24] Goethert HK, Telford SR 3rd (2009) Nonrandom distribution of vector ticks (*Dermacentor variabilis*) infected by *Francisella tularensis* . PLoS Pathogens 5: e1000319. 19247435 10.1371/journal.ppat.1000319 PMC2642597

[25] Goethert HK, Telford SR 3rd (2011) Differential mortality of dog tick vectors due to infection by diverse *Francisella tularensis tularensis* genotypes. Vector Borne and Zoonotic Diseases 11: 1263–1268. 21612530 10.1089/vbz.2010.0237 PMC3162643

[26] Champion MD, Zeng Q, Nix EB, Nano FE, Keim P, et al (2009) Comparative genomic characterization of *Francisella tularensis* strains belonging to low and high virulence subspecies. PLoS Pathogens 5: e1000459. 19478886 10.1371/journal.ppat.1000459 PMC2682660

[27] Berrada ZL, Telford SR 3rd (2010) Diversity of *Francisella* species in environmental samples from Martha's Vineyard, Massachusetts. Microbial Ecology 59: 277–283. 19669828 10.1007/s00248-009-9568-y PMC2836248

[28] Johansson A, Göransson I, Larsson P, Sjöstedt A (2001) Extensive allelic variation among *Francisella tularensis* strains in a short-sequence tandem repeat region. Journal of Clinical Microbiology 39: 3140–3146. 11526142 10.1128/JCM.39.9.3140-3146.2001 PMC88310

[29] Reif KE, Palmer GH, Ueti MW, Scoles GA, Margolis JJ, et al (2011) *Dermacentor andersoni* transmission of *Francisella tularensis* subsp. *novicida* reflects bacterial colonization, dissemination, and replication coordinated with tick feeding. Infection and Immunity 79: 4941–4946. 21930762 10.1128/IAI.05676-11 PMC3232653

[30] Narasimhan S, Rajeevan N, Liu L, Zhao YO, Heisig J, et al (2014) Gut Microbiota of the Tick Vector *Ixodes scapularis* Modulate Colonization of the Lyme Disease Spirochete. Cell Host & Microbe 15: 58–71. 24439898 10.1016/j.chom.2013.12.001 PMC3905459

[31] Ueti MW, Reagan JO Jr, Knowles DP Jr, Scoles GA, Shkap V, et al (2007) Identification of midgut and salivary glands as specific and distinct barriers to efficient tick-borne transmission of *Anaplasma marginale* . Infection and Immunity 75: 2959–2964. 17420231 10.1128/IAI.00284-07 PMC1932854

[32] Taylor LH, Walliker D, Read AF (1997) Mixed-genotype infections of the rodent malaria *Plasmodium chabaudi* are more infectious to mosquitoes than single-genotype infections. Parasitology 115 (Pt 2) 121–132. 10190168 10.1017/s0031182097001145

[33] Taylor LH, Walliker D, Read AF (1997) Mixed-genotype infections of malaria parasites: within-host dynamics and transmission success of competing clones. Proceedings Biological Sciences/The Royal Society 264: 927–935. 10.1098/rspb.1997.0128 PMC1688430 9225482

[34] de Roode JC, Helinski ME, Anwar MA, Read AF (2005) Dynamics of multiple infection and within-host competition in genetically diverse malaria infections. The American Naturalist 166: 531–542. 10.1086/491659 16224719

[35] de Roode JC, Pansini R, Cheesman SJ, Helinski ME, Huijben S, et al (2005) Virulence and competitive ability in genetically diverse malaria infections. Proceedings of the National Academy of Sciences of the United States of America 102: 7624–7628. 15894623 10.1073/pnas.0500078102 PMC1140419

[36] Brackney DE, Pesko KN, Brown IK, Deardorff ER, Kawatachi J, et al (2011) West Nile virus genetic diversity is maintained during transmission by *Culex pipiens quinquefasciatus* mosquitoes. PLoS One 6: e24466. 21935412 10.1371/journal.pone.0024466 PMC3171416

[37] Ciota AT, Ehrbar DJ, Van Slyke GA, Payne AF, Willsey GG, et al (2012) Quantification of intrahost bottlenecks of West Nile virus in *Culex pipiens* mosquitoes using an artificial mutant swarm. Infection, Genetics and Evolution 12: 557–564. 10.1016/j.meegid.2012.01.022 PMC3314143 22326536

[38] Eswarappa SM, Estrela S, Brown SP (2012) Within-host dynamics of multi-species infections: facilitation, competition and virulence. PloS One 7: e38730. 22737220 10.1371/journal.pone.0038730 PMC3380906

[39] Joseph SB, Swanstrom R (2014) HIV/AIDS. A fitness bottleneck in HIV-1 transmission. Science 345: 136–137. 25013044 10.1126/science.1257425

[40] Carlson JM, Schaefer M, Monaco DC, Batorsky R, Claiborne DT, et al (2014) HIV transmission. Selection bias at the heterosexual HIV-1 transmission bottleneck. Science 345: 1254031. 25013080 10.1126/science.1254031 PMC4289910

[41] Rego RO, Bestor A, Stefka J, Rosa PA (2014) Population bottlenecks during the infectious cycle of the Lyme disease spirochete Borrelia burgdorferi. PloS One 9: e101009. 24979342 10.1371/journal.pone.0101009 PMC4076273

[42] Jerzak GV, Brown I, Shi PY, Kramer LD, Ebel GD (2008) Genetic diversity and purifying selection in West Nile virus populations are maintained during host switching. Virology 374: 256–260. 18395240 10.1016/j.virol.2008.02.032 PMC2409985

[43] Forrester NL, Guerbois M, Seymour RL, Spratt H, Weaver SC (2012) Vector-borne transmission imposes a severe bottleneck on an RNA virus population. PLoS Pathogens 8: e1002897. 23028310 10.1371/journal.ppat.1002897 PMC3441635

[44] Dark MJ, Herndon DR, Kappmeyer LS, Gonzales MP, Nordeen E, et al (2009) Conservation in the face of diversity: multistrain analysis of an intracellular bacterium. BMC Genomics 10: 16. 19134224 10.1186/1471-2164-10-16 PMC2649000

[45] Herndon DR, Ueti MW, Reif KE, Noh SM, Brayton KA, et al (2013) Identification of multilocus genetic heterogeneity in *Anaplasma marginale* subsp. central and its restriction following tick-borne transmission. Infection and Immunity 81: 1852–1858. 23509140 10.1128/IAI.00199-13 PMC3648015

[46] Rodriquez JL, Palmer GH, Knowles DP Jr, Brayton KA (2005) Distinctly different *msp2* pseudogene repertoires in *Anaplasma marginale* strains that are capable of superinfection. Gene 361: 127–132. 16202540 10.1016/j.gene.2005.06.038

[47] Tettelin H, Masignani V, Cieslewicz MJ, Donati C, Medini D, et al (2005) Genome analysis of multiple pathogenic isolates of Streptococcus agalactiae: implications for the microbial “pan-genome”. Proceedings of the National Academy of Sciences of the United States of America 102: 13950–13955. 16172379 10.1073/pnas.0506758102 PMC1216834

[48] Faburay B, Jongejan F, Taoufik A, Ceesay A, Geysen D (2008) Genetic diversity of *Ehrlichia ruminantium* in *Amblyomma variegatum* ticks and small ruminants in The Gambia determined by restriction fragment profile analysis. Veterinary Microbiology 126: 189–199. 17646061 10.1016/j.vetmic.2007.06.010

[49] Znazen A, Khrouf F, Elleuch N, Lahiani D, Marrekchi C, et al (2013) Multispacer typing of *Rickettsia* isolates from humans and ticks in Tunisia revealing new genotypes. Parasites & Vectors 6: 367. 24380581 10.1186/1756-3305-6-367 PMC3883474

[50] Futse JE, Brayton KA, Dark MJ, Knowles DP Jr, Palmer GH (2008) Superinfection as a driver of genomic diversification in antigenically variant pathogens. Proceedings of the National Academy of Sciences of the United States of America 105: 2123–2127. 18252822 10.1073/pnas.0710333105 PMC2538888

[51] Gallagher LA, Ramage E, Jacobs MA, Kaul R, Brittnacher M, et al (2007) A comprehensive transposon mutant library of *Francisella novicida*, a bioweapon surrogate. Proceedings of the National Academy of Sciences of the United States of America 104: 1009–1014. 17215359 10.1073/pnas.0606713104 PMC1783355

