# Supplementary figures and images for: Restriction of Francisella novicida Genetic Diversity during Infection of the Vector Midgut
Source: PLoS Pathog. 2014 Nov 13;10(11):e1004499. doi: 10.1371/journal.ppat.1004499 (PMC4231110; doi:10.1371/journal.ppat.1004499)

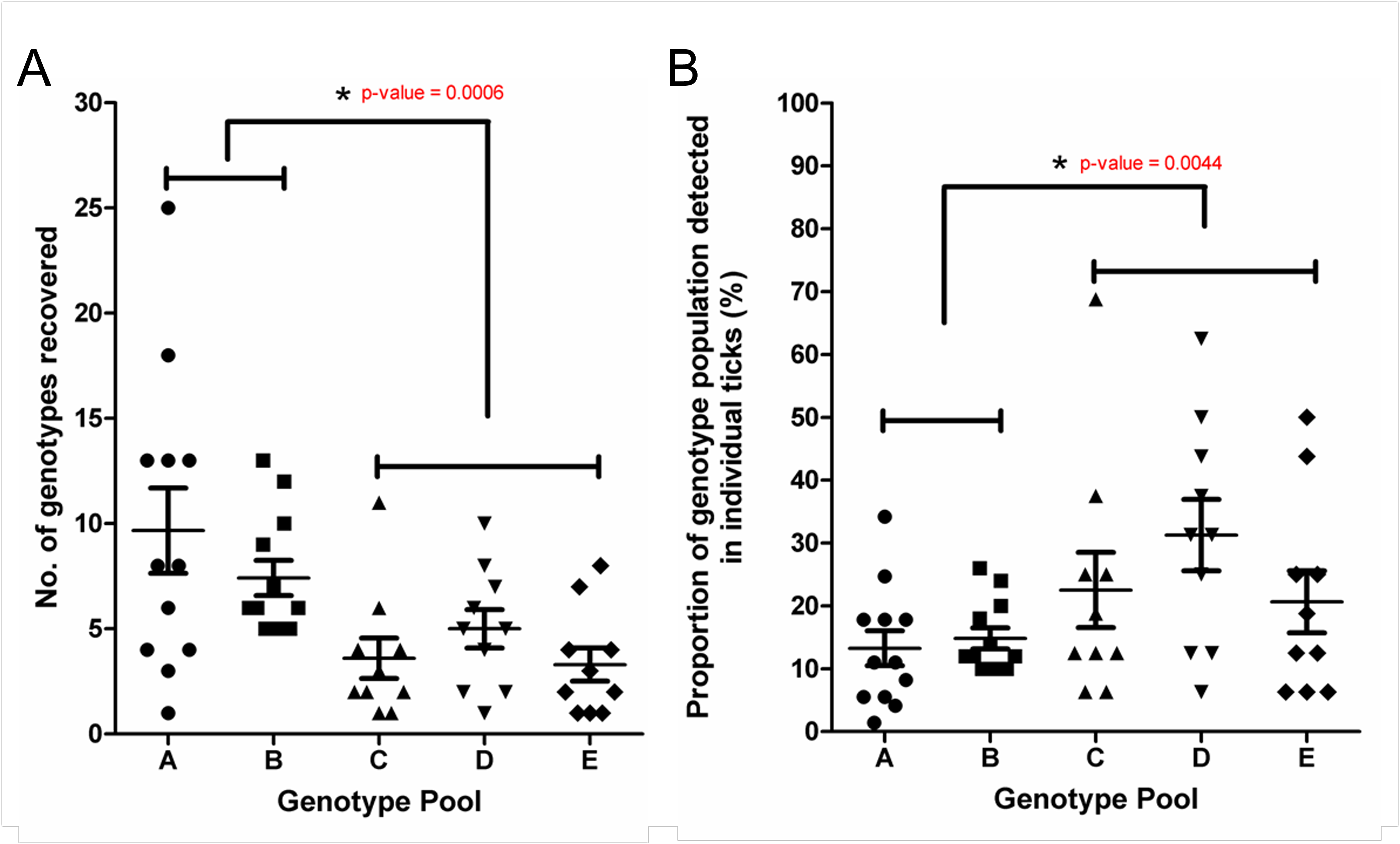

Supplement: Figure S1 — Number and proportional recovery of genotypes from ticks exposed to genotype populations varying in diversity. Comparison of the mean genotype recovery in individual ticks that fed upon mice inoculated with large- or small- genotype pools as the (A) number of genotypes recovered and the (B) proportion of available genotypes recovered. A significantly greater number of genotypes were recovered in large-genotype pools compared to small-genotype pools (t = 3.783, P = 0.0006); however, a significantly greater proportion of the total available genotypes were recovered in small-genotype pools compared to large-genotype pools (t = 3.011, P = 0.0044). (TIF) [file ppat.1004499.s001.tif]

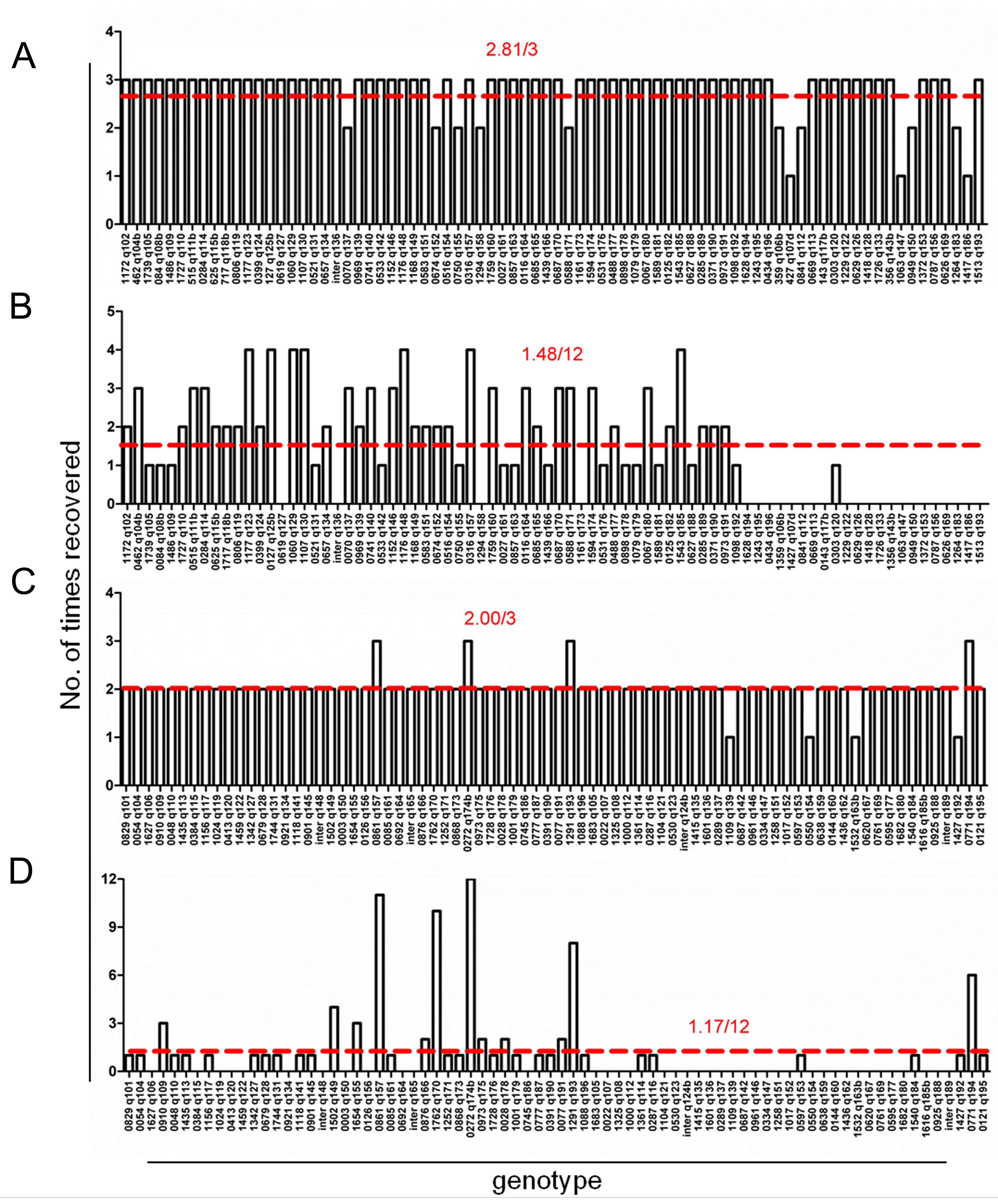

Supplement: Figure S2 — Recovery of individual genotypes from mice and ticks exposed to large-genotype pools. Frequency of individual genotype recovery from (A) mice and (B) ticks exposed to Pool A genotypes. Frequency of individual genotype recovery from (C) mice and (D) ticks exposed to Pool B genotypes. The value included above each graph and the red dashed line indicates the mean number of times a single genotype was recovered from mice and ticks. A significantly higher proportion of genotypes were recovered from mice compared with ticks (χ2 = 501.8, P<0.0001). (TIF) [file ppat.1004499.s002.tif]

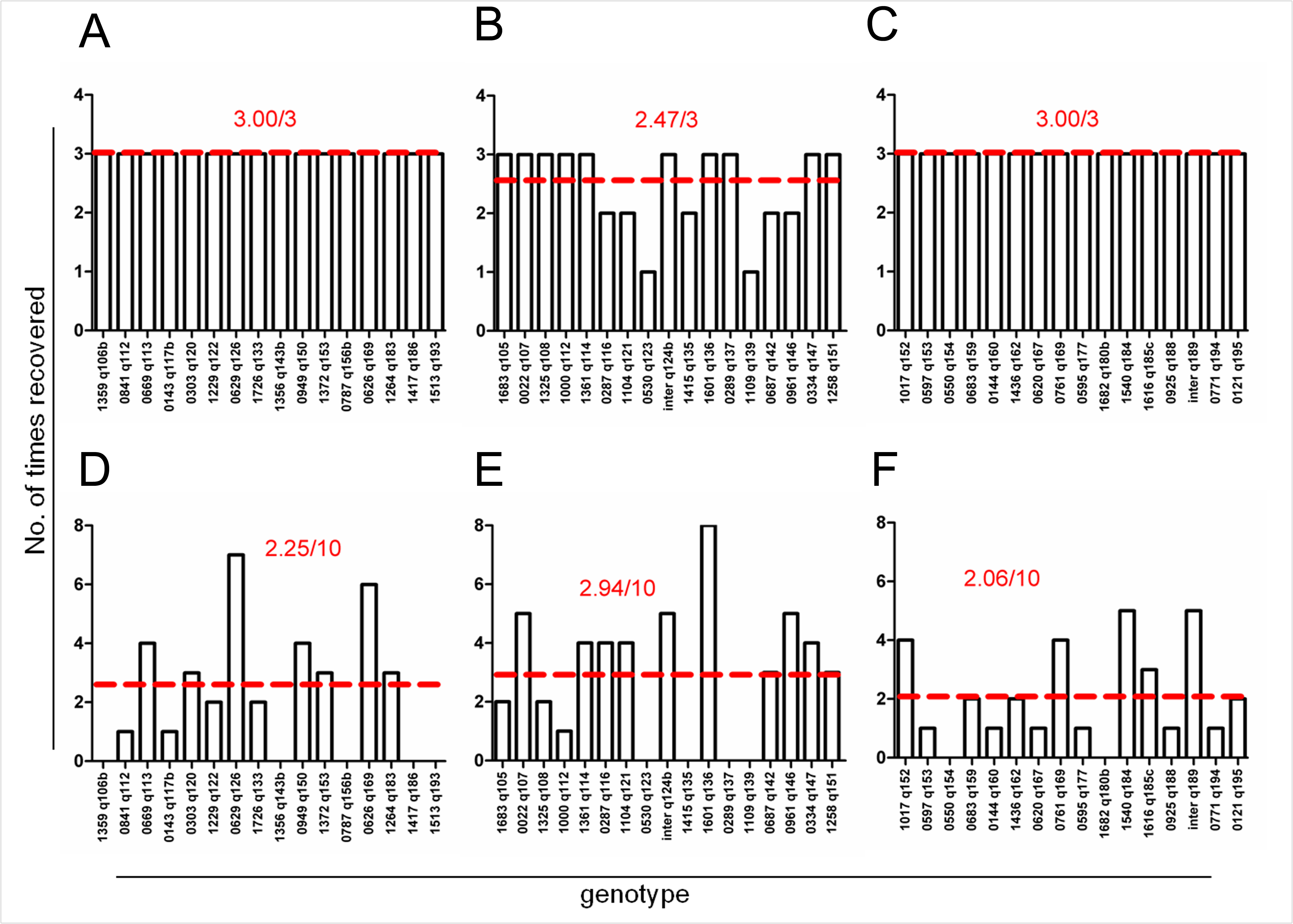

Supplement: Figure S3 — Recovery of individual genotypes from mice and ticks exposed to small-genotype pools. Frequency of individual genotype recovery from mice, (A) Pool C, (B) Pool D, (C) Pool E. Frequency of individual genotype recovery from ticks, (D) Pool C, (E) Pool D, (F) Pool E. The value above each graph and the red dashed line indicates the mean number of times a single genotype was recovered from mice and ticks. A significantly higher proportion of genotypes were recovered from mice compared with ticks (χ2 = 267.0, P<0.0001). (TIF) [file ppat.1004499.s003.tif]

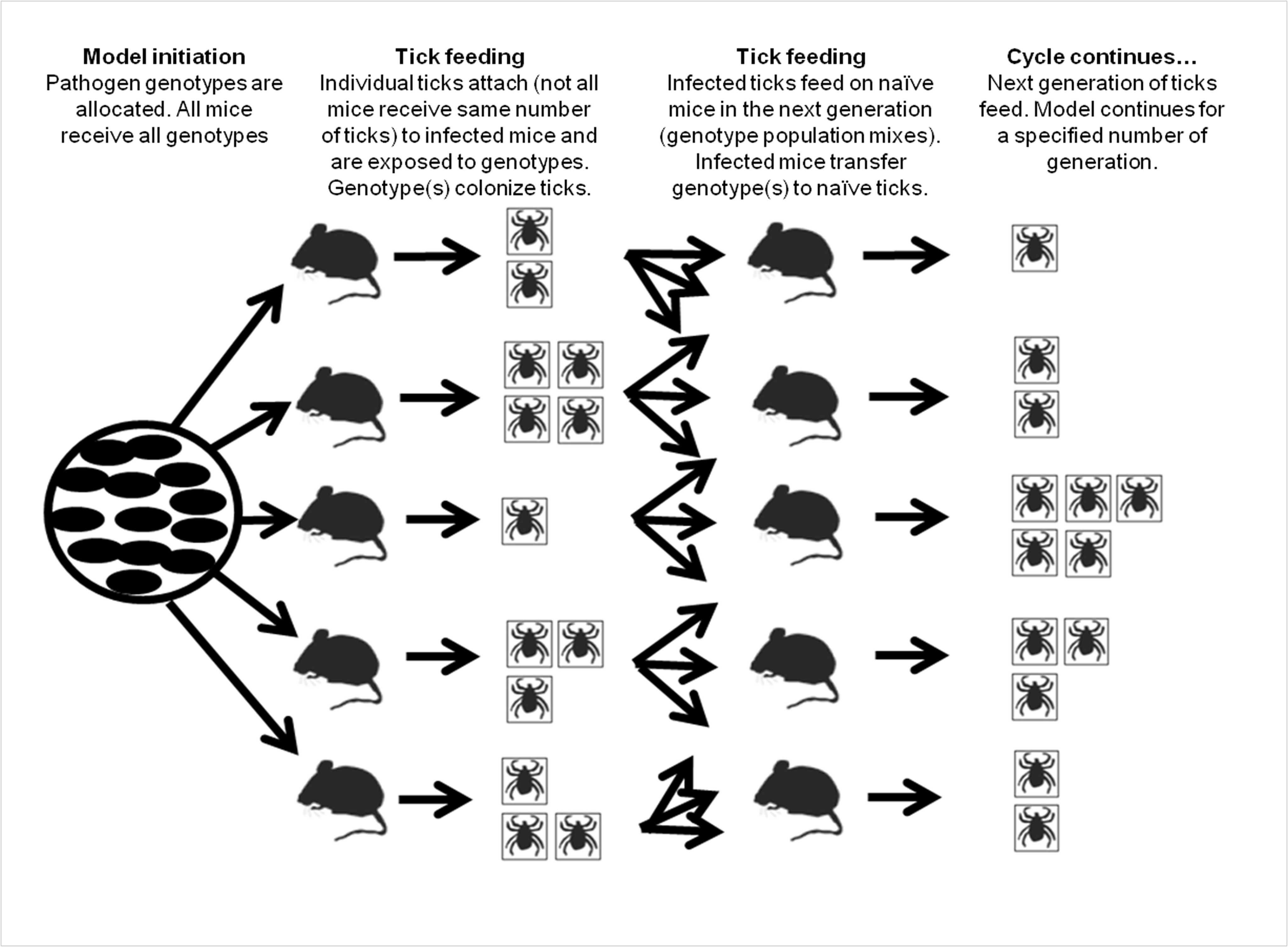

Supplement: Figure S4 — Flowchart of the population model. Shown are the first two generations of the model. Lines represent feeding events and/or pathogen transfer events. To initiate the model, all pathogen genotypes are allocated to all mice. These mice are fed upon by ticks, with all ticks finding a host (not all mice are fed upon by the same number of ticks), and pathogen genotypes are acquire by feeding ticks. Infected ticks then feed on naïve mice in the next generation, mixing the tick population and resulting in transmission. These mice are then fed upon by second generation naïve ticks. The cycle continues for a specified number of generations. (TIF) [file ppat.1004499.s004.tif]

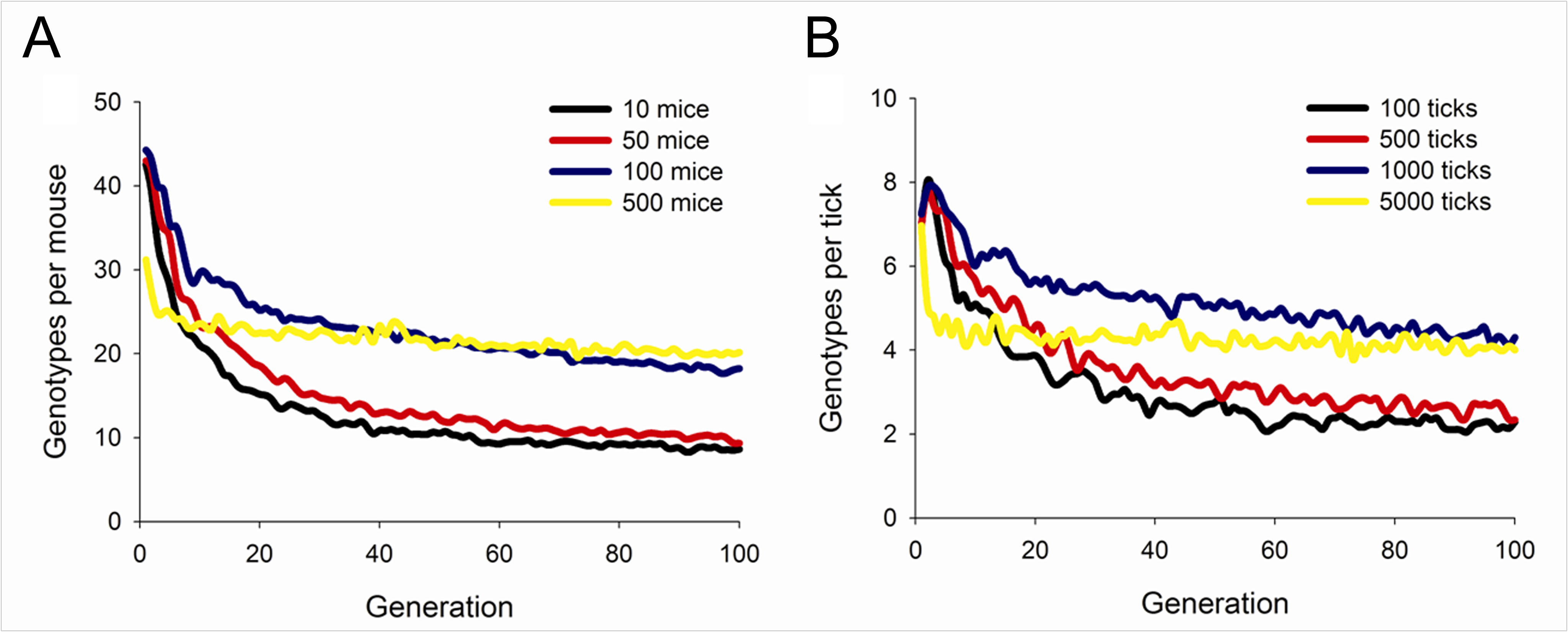

Supplement: Figure S5 — Retention of genotype diversity as a function of varying host and tick abundance. Number of pathogen genotypes per (A) mouse and (B) tick in simulations with varying abundances of mice and ticks. In all simulations the vector-to-host ratio was 10∶1, but the number of vectors and hosts was varied. (TIF) [file ppat.1004499.s005.tif]

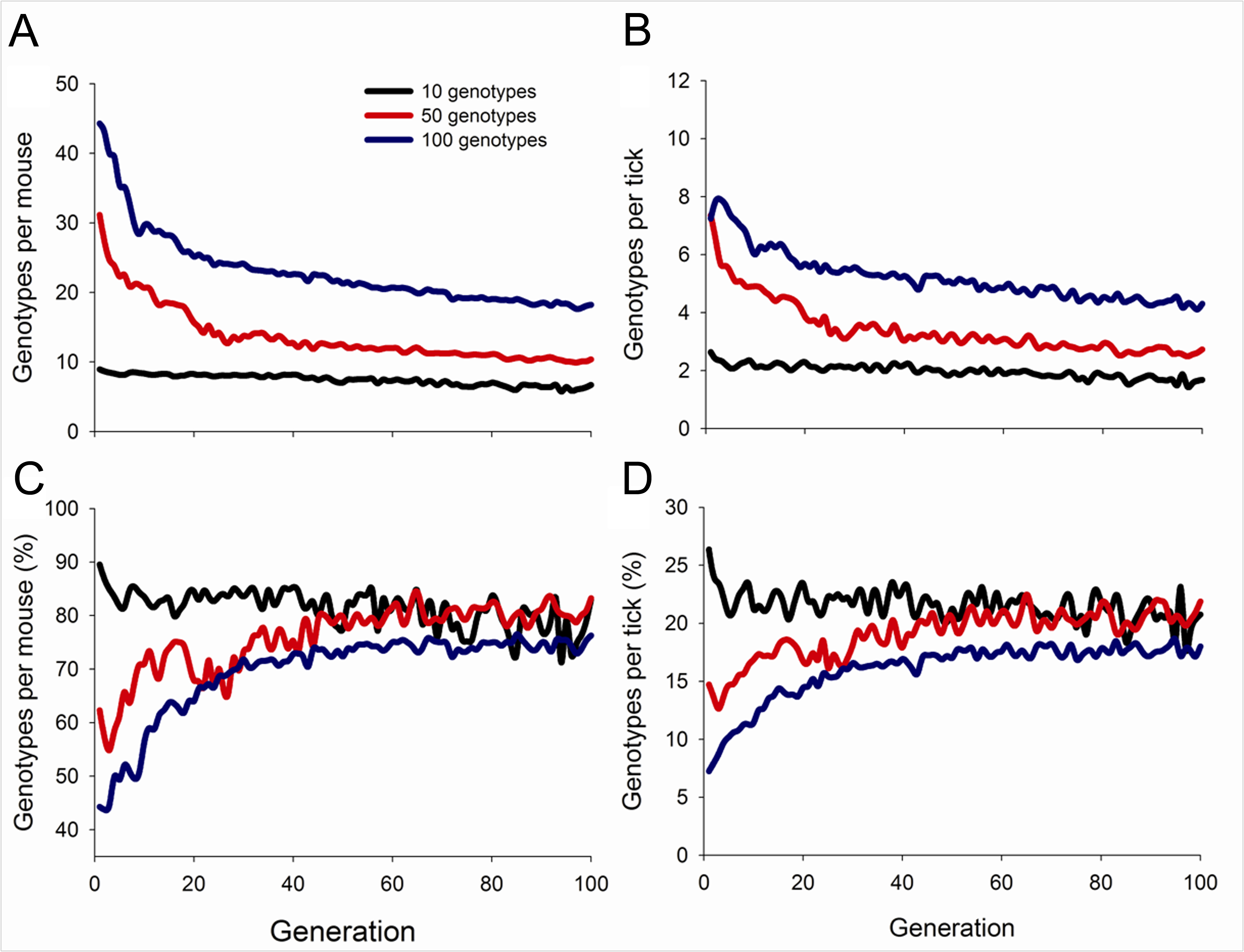

Supplement: Figure S6 — Retention of genotype diversity in individual mice and ticks as a function of the number of genotypes in a population. Number of pathogen genotypes per (A) mouse and (B) tick, and the proportion of pathogen genotypes per (C) mouse and (D) tick, in simulations with varying initial number of pathogen genotypes. In all simulations the vector-to-host ratio was 10∶1 with 100 mice and 1000 ticks, but the number of initial pathogen genotypes was varied. (TIF) [file ppat.1004499.s006.tif]
