# Supplementary material for: Restriction of Francisella novicida Genetic Diversity during Infection of the Vector Midgut
Source: PLoS Pathog. 2014 Nov 13;10(11):e1004499. doi: 10.1371/journal.ppat.1004499 (PMC4231110; doi:10.1371/journal.ppat.1004499)
Supplement: Table S1 — F. novicida transposon mutants used in this study. (PDF) [file ppat.1004499.s007.pdf]

F. novicida transposon mutant	*F. novicida* locus tag	Pool A	Pool B	Pool C	Pool D	Pool E	Individual
tnfn1_pw060510p03q102	FTN_1172	X					
tnfn1_pw060510p03q103	FTN_0526	X					
tnfn1_pw060510p03q104	FTN_0462	X					
tnfn1_pw060510p03q105	FTN_1739	X					
tnfn1_pw060510p03q106	FTN_1359	X		X			
tnfn1_pw060510p03q107	FTN_1427	X					
tnfn1_pw060510p03q108	FTN_0084	X					
tnfn1_pw060510p03q109	FTN_1486	X					
tnfn1_pw060510p03q110	FTN_1727	X					
tnfn1_pw060510p03q111	FTN_0515	X					
tnfn1_pw060510p03q112	FTN_0841	X		X			
tnfn1_pw060510p03q113	FTN_0669	X		X			
tnfn1_pw060510p03q114	FTN_0284	X					
tnfn1_pw060510p03q115	FTN_0625	X					
tnfn1_pw060510p03q116	intergenic	X					
tnfn1_pw060510p03q117	FTN_0143	X		X			
tnfn1_pw060510p03q118	FTN_1717	X					
tnfn1_pw060510p03q119	FTN_0806	X					
tnfn1_pw060510p03q120	FTN_0303	X		X			
tnfn1_pw060510p03q121	FTN_1699	X					
tnfn1_pw060510p03q122	FTN_1229	X		X			
tnfn1_pw060510p03q123	FTN_1177	X					
tnfn1_pw060510p03q124	FTN_0399	X					
tnfn1_pw060510p03q125	FTN_0127	X					
tnfn1_pw060510p03q126	FTN_0629	X		X			
tnfn1_pw060510p03q127	FTN_0619	X					
tnfn1_pw060510p03q128	FTN_1418	X					
tnfn1_pw060510p03q129	FTN_1060	X					
tnfn1_pw060510p03q130	FTN_1107	X					
tnfn1_pw060510p03q131	FTN_0521	X					
tnfn1_pw060510p03q132	FTN_1463	X		X			
tnfn1_pw060510p03q133	FTN_1726	X					
tnfn1_pw060510p03q134	FTN_0657	X					
tnfn1_pw060510p03q135	FTN_1254	X					
tnfn1_pw060510p03q136	intergenic	X					
tnfn1_pw060510p03q137	FTN_0070	X					
tnfn1_pw060510p03q139	FTN_0969	X					
tnfn1_pw060510p03q140	FTN_0741	X					
tnfn1_pw060510p03q141	FTN_1493	X					
tnfn1_pw060510p03q142	FTN_0533	X					
tnfn1_pw060510p03q143	FTN_1356	X		X			Genotype 1
tnfn1_pw060510p03q144	FTN_0171	X					
tnfn1_pw060510p03q145	FTN_1309	X					
tnfn1_pw060510p03q146	FTN_1152	X					
tnfn1_pw060510p03q147	FTN_1063	X					
tnfn1_pw060510p03q148	FTN_1176	X					
tnfn1_pw060510p03q149	FTN_1168	X					
tnfn1_pw060510p03q150	FTN_0949	X		X			
tnfn1_pw060510p03q151	FTN_0583	X					
tnfn1_pw060510p03q152	FTN_0674	X					
tnfn1_pw060510p03q153	FTN_1372	X		X			
tnfn1_pw060510p03q154	FTN_0516	X					

tnfn1_pw060510p03q155	FTN_0750	X				
tnfn1_pw060510p03q156	FTN_0787	X		X		Genotype 2
tnfn1_pw060510p03q157	FTN_0316	X				
tnfn1_pw060510p03q158	FTN_1294	X				
tnfn1_pw060510p03q159	FTN_1051	X				
tnfn1_pw060510p03q160	FTN_1759	X				
tnfn1_pw060510p03q161	FTN_0027	X				
tnfn1_pw060510p03q162	FTN_0177	X				
tnfn1_pw060510p03q163	FTN_0857	X				
tnfn1_pw060510p03q164	FTN_0116	X				
tnfn1_pw060510p03q165	FTN_0685	X				
tnfn1_pw060510p03q166	FTN_1439	X				
tnfn1_pw060510p03q168	FTN_0598	X				
tnfn1_pw060510p03q169	FTN_0626	X		X		
tnfn1_pw060510p03q170	FTN_0687	X				
tnfn1_pw060510p03q171	FTN_0588	X				
tnfn1_pw060510p03q172	FTN_0527	X				
tnfn1_pw060510p03q173	FTN_1161	X				
tnfn1_pw060510p03q174	FTN_1594	X				
tnfn1_pw060510p03q175	FTN_1732	X				
tnfn1_pw060510p03q176	FTN_0531	X				
tnfn1_pw060510p03q177	FTN_0488	X				
tnfn1_pw060510p03q178	FTN_0898	X				
tnfn1_pw060510p03q179	FTN_1079	X				
tnfn1_pw060510p03q180	FTN_0067	X				
tnfn1_pw060510p03q181	FTN_1589	X				
tnfn1_pw060510p03q182	FTN_0125	X				
tnfn1_pw060510p03q183	FTN_1264	X		X		
tnfn1_pw060510p03q184	FTN_1715	X				
tnfn1_pw060510p03q185	FTN_1543	X				
tnfn1_pw060510p03q186	FTN_1417	X		X		Genotype 3
tnfn1_pw060510p03q187	FTN_0178	X				
tnfn1_pw060510p03q188	FTN_0627	X				
tnfn1_pw060510p03q189	FTN_0285	X				
tnfn1_pw060510p03q190	FTN_0371	X				
tnfn1_pw060510p03q191	FTN_0973	X				
tnfn1_pw060510p03q192	FTN_1098	X				
tnfn1_pw060510p03q193	FTN_1513	X		X		
tnfn1_pw060510p03q194	FTN_1628	X				
tnfn1_pw060510p03q195	FTN_1243	X				
tnfn1_pw060510p03q196	FTN_0434	X				
tnfn1_pw060419p04q101	FTN_0829		X			
tnfn1_pw060419p04q102	FTN_1129		X			
tnfn1_pw060419p04q103	FTN_0183		X			
tnfn1_pw060419p04q104	FTN_0054		X			
tnfn1_pw060419p04q105	FTN_1683		X		X	
tnfn1_pw060419p04q106	FTN_1627		X			
tnfn1_pw060419p04q107	FTN_0022		X		X	
tnfn1_pw060419p04q108	FTN_1325		X		X	
tnfn1_pw060419p04q109	FTN_0910		X			
tnfn1_pw060419p04q110	FTN_0048		X			
tnfn1_pw060419p04q111	FTN_0580		X			
tnfn1_pw060419p04q112	FTN_1000		X		X	

tnfn1_pw060419p04q113	FTN_1435	X			
tnfn1_pw060419p04q114	FTN_1361	X	X		
tnfn1_pw060419p04q115	FTN_0384	X			
tnfn1_pw060419p04q116	FTN_0287	X	X		
tnfn1_pw060419p04q117	FTN_1156	X			
tnfn1_pw060419p04q118	FTN_1172	X			
tnfn1_pw060419p04q119	FTN_1024	X			
tnfn1_pw060419p04q120	FTN_0413	X			
tnfn1_pw060419p04q121	FTN_1104	X	X		
tnfn1_pw060419p04q122	FTN_1459	X			
tnfn1_pw060419p04q123	FTN_0530	X	X		Genotype 4
tnfn1_pw060419p04q124	intergenic	X	X		
tnfn1_pw060419p04q125	FTN_1050	X			
tnfn1_pw060419p04q126	FTN_0296	X			
tnfn1_pw060419p04q127	FTN_1342	X			
tnfn1_pw060419p04q128	FTN_0679	X			
tnfn1_pw060419p04q129	FTN_1290	X			
tnfn1_pw060419p04q130	FTN_1585	X			
tnfn1_pw060419p04q131	FTN_1744	X			
tnfn1_pw060419p04q132	FTN_1282	X			
tnfn1_pw060419p04q134	FTN_0921	X			
tnfn1_pw060419p04q135	FTN_1415	X	X		
tnfn1_pw060419p04q136	FTN_1601	X	X		
tnfn1_pw060419p04q137	FTN_0289	X	X		
tnfn1_pw060419p04q138	FTN_1470	X			
tnfn1_pw060419p04q139	FTN_1109	X	X		Genotype 5
tnfn1_pw060419p04q140	FTN_1465	X			
tnfn1_pw060419p04q141	FTN_1118	X			
tnfn1_pw060419p04q142	FTN_0687	X	X		
tnfn1_pw060419p04q143	FTN_0386	X			
tnfn1_pw060419p04q144	FTN_0426	X			
tnfn1_pw060419p04q145	FTN_0901	X			
tnfn1_pw060419p04q146	FTN_0961	X	X		
tnfn1_pw060419p04q147	FTN_0334	X	X		
tnfn1_pw060419p04q148	intergenic	X			
tnfn1_pw060419p04q149	FTN_1502	X			
tnfn1_pw060419p04q150	FTN_0003	X			
tnfn1_pw060419p04q151	FTN_1258	X	X		
tnfn1_pw060419p04q152	FTN_1017	X		X	
tnfn1_pw060419p04q153	FTN_0597	X		X	
tnfn1_pw060419p04q154	FTN_0550	X		X	
tnfn1_pw060419p04q155	FTN_1654	X			
tnfn1_pw060419p04q156	FTN_0126	X			
tnfn1_pw060419p04q157	FTN_0861	X			
tnfn1_pw060419p04q158	FTN_1430	X			
tnfn1_pw060419p04q159	FTN_0638	X		X	
tnfn1_pw060419p04q160	FTN_0144	X		X	
tnfn1_pw060419p04q161	FTN_0085	X			
tnfn1_pw060419p04q162	FTN_1436	X		X	
tnfn1_pw060419p04q163	FTN_1532	X			
tnfn1_pw060419p04q164	FTN_0692	X			
tnfn1_pw060419p04q165	intergnic	X			
tnfn1_pw060419p04q166	FTN_0876	X			

tnfn1_pw060419p04q167	FTN_0620	X	X	
tnfn1_pw060419p04q168	FTN_1091	X		
tnfn1_pw060419p04q169	FTN_0761	X	X	
tnfn1_pw060419p04q170	FTN_1762	X		
tnfn1_pw060419p04q171	FTN_1252	X		
tnfn1_pw060419p04q172	FTN_0757	X		
tnfn1_pw060419p04q173	FTN_0868	X		
tnfn1_pw060419p04q174	FTN_0272	X		
tnfn1_pw060419p04q175	FTN_0973	X		
tnfn1_pw060419p04q176	FTN_1728	X		
tnfn1_pw060419p04q177	FTN_0595	X	X	
tnfn1_pw060419p04q178	FTN_0028	X		
tnfn1_pw060419p04q179	FTN_1001	X		
tnfn1_pw060419p04q180	FTN_1682	X	X	Genotype 6
tnfn1_pw060419p04q182	FTN_1716	X		
tnfn1_pw060419p04q183	FTN_1240	X		
tnfn1_pw060419p04q184	FTN_1540	X	X	
tnfn1_pw060419p04q185	FTN_1616	X	X	
tnfn1_pw060419p04q186	FTN_0745	X		
tnfn1_pw060419p04q187	FTN_0777	X		
tnfn1_pw060419p04q188	FTN_0925	X	X	
tnfn1_pw060419p04q189	intergenic	X	X	
tnfn1_pw060419p04q190	FTN_0391	X		
tnfn1_pw060419p04q191	FTN_0077	X		
tnfn1_pw060419p04q192	FTN_1427	X		
tnfn1_pw060419p04q193	FTN_1291	X		
tnfn1_pw060419p04q194	FTN_0771	X	X	
tnfn1_pw060419p04q195	FTN_0121	X	X	
tnfn1_pw060419p04q196	FTN_1088	X		
