# Supplementary material for: Restriction of Francisella novicida Genetic Diversity during Infection of the Vector Midgut
Source: PLoS Pathog. 2014 Nov 13;10(11):e1004499. doi: 10.1371/journal.ppat.1004499 (PMC4231110; doi:10.1371/journal.ppat.1004499)
Supplement: Table S2 — List of primers used in this study. (PDF) [file ppat.1004499.s008.pdf]

F. novicida transposon mutant	*F. novicida* locus tag	Mutant specific primer
tnfn1_pw060510p03q102	FTN_1172	gatttgattccaaggattatctc
tnfn1_pw060510p03q103	FTN_0526	cctttgtcactcatagcacttat
tnfn1_pw060510p03q104	FTN_0462	agaaagtataaaggtgcgaattt
tnfn1_pw060510p03q105	FTN_1739	tttcatcaataaagtcagcaaac
tnfn1_pw060510p03q106	FTN_1359	atatctatcatcatcgcgtttg
tnfn1_pw060510p03q107	FTN_1427	ctttcattattcggccaag
tnfn1_pw060510p03q108	FTN_0084	tatgaaattagactccatcaagc
tnfn1_pw060510p03q109	FTN_1486	gttatttagttgattgggcaaag
tnfn1_pw060510p03q110	FTN_1727	atgttgagctttcaggatcttat
tnfn1_pw060510p03q111	FTN_0515	cgtattgtgtttaggttatctgg
tnfn1_pw060510p03q112	FTN_0841	taagacgtggttctcaaactaaa
tnfn1_pw060510p03q113	FTN_0669	catcatatttgtgaaagattgct
tnfn1_pw060510p03q114	FTN_0284	ctgaattactcattagcctctcc
tnfn1_pw060510p03q115	FTN_0625	ctcatcttgattcttaaatggag
tnfn1_pw060510p03q116	intergenic	taaatacaggtgagctaataccg
tnfn1_pw060510p03q117	FTN_0143	gtcttaggagctgaaattgactt
tnfn1_pw060510p03q118	FTN_1717	cttccagatagtgcaacgac
tnfn1_pw060510p03q119	FTN_0806	ggtcttactcagataatcccaat
tnfn1_pw060510p03q120	FTN_0303	atttgagtttacctttagcgact
tnfn1_pw060510p03q121	FTN_1699	tttatcttgctcttgaccatcta
tnfn1_pw060510p03q122	FTN_1229	tttaggtgatttcgtatgctatg
tnfn1_pw060510p03q123	FTN_1177	agagctgatttgtttccaatagt
tnfn1_pw060510p03q124	FTN_0399	tcctatatgtcagtggtgaagag
tnfn1_pw060510p03q125	FTN_0127	gattcaagtgttattggcataga
tnfn1_pw060510p03q126	FTN_0629	tcacaaactctactctcatgctc
tnfn1_pw060510p03q127	FTN_0619	aaagtctgtctaacggtcatagc
tnfn1_pw060510p03q128	FTN_1418	agcataattgtatttggatcatt
tnfn1_pw060510p03q129	FTN_1060	gaagcggttaaatcaggtaaata
tnfn1_pw060510p03q130	FTN_1107	tttcattgtcatttaaggctcta
tnfn1_pw060510p03q131	FTN_0521	tctctatgtttagtatcggtcca
tnfn1_pw060510p03q132	FTN_1463	tcatgtaagccttgttgtgata
tnfn1_pw060510p03q133	FTN_1726	tgcattccgattatagacatatt
tnfn1_pw060510p03q134	FTN_0657	gcgaatgagtaatttattgtttg
tnfn1_pw060510p03q135	FTN_1254	tttgtttatattctccagttgaca
tnfn1_pw060510p03q136	intergenic	catttagttggttagacattatcaa
tnfn1_pw060510p03q137	FTN_0070	cctataaagatgctgaaatgaca
tnfn1_pw060510p03q139	FTN_0969	ctttaatataacgccaaatacga
tnfn1_pw060510p03q140	FTN_0741	agcatactaagattattcctggag
tnfn1_pw060510p03q141	FTN_1493	acacctttatcattcttggttaaa
tnfn1_pw060510p03q142	FTN_0533	aaattatagaagaagcgccaata
tnfn1_pw060510p03q143	FTN_1356	gctatttcattctcataattcca
tnfn1_pw060510p03q144	FTN_0171	ggtaggtcatacttgtttaatgct
tnfn1_pw060510p03q145	FTN_1309	ttgatttgggcttaatagaaagt
tnfn1_pw060510p03q146	FTN_1152	actaaatcctttgaaatgggaat
tnfn1_pw060510p03q147	FTN_1063	atttgaggatgtcttagcagaat
tnfn1_pw060510p03q148	FTN_1176	gagaatgccttagtgatagttga
tnfn1_pw060510p03q149	FTN_1168	aaatcttgtagtttatccgcttc
tnfn1_pw060510p03q150	FTN_0949	tagagagtaagtcgtttcagcag

tnfn1_pw060510p03q151	FTN_0583	tattccatttccattaagacaca
tnfn1_pw060510p03q152	FTN_0674	ggaatctttatcagcattacaag
tnfn1_pw060510p03q153	FTN_1372	ttcagacatatacagcctttgat
tnfn1_pw060510p03q154	FTN_0516	ccaactttgtgaactaatggtaa
tnfn1_pw060510p03q155	FTN_0750	cctgtatcaaggtatgcaagtt
tnfn1_pw060510p03q156	FTN_0787	tgcatacctaaactcagcttg
tnfn1_pw060510p03q157	FTN_0316	ttgtgttgctagagaaagaattt
tnfn1_pw060510p03q158	FTN_1294	ttaacttctttgaatcagcaaga
tnfn1_pw060510p03q159	FTN_1051	gttaaatcagcattaagttcacg
tnfn1_pw060510p03q160	FTN_1759	tcatataaatcgctccacctaat
tnfn1_pw060510p03q161	FTN_0027	ctttgatgatggtgcatatagag
tnfn1_pw060510p03q162	FTN_0177	gcgctctatctgtaacaacttta
tnfn1_pw060510p03q163	FTN_0857	gaatttgctataataaccgccta
tnfn1_pw060510p03q164	FTN_0116	catattaagtgaagaggaacctg
tnfn1_pw060510p03q165	FTN_0685	tatgcagagtcagctaaattatg
tnfn1_pw060510p03q166	FTN_1439	aatcgttactgtagctctggatt
tnfn1_pw060510p03q168	FTN_0598	gttaaatgtcgtattggctatga
tnfn1_pw060510p03q169	FTN_0626	ctttgttggttagcttgtaattg
tnfn1_pw060510p03q170	FTN_0687	ttgatttaagtgttgagcaaagt
tnfn1_pw060510p03q171	FTN_0588	aatatcaactcagcctgtttctt
tnfn1_pw060510p03q172	FTN_0527	aaataatgttgccacgatgt
tnfn1_pw060510p03q173	FTN_1161	atggcttaaattatcagatgttg
tnfn1_pw060510p03q174	FTN_1594	tttagcatttgagcgaattatta
tnfn1_pw060510p03q175	FTN_1732	ttaccattcgctgctattactac
tnfn1_pw060510p03q176	FTN_0531	tgccttactgtttcatataatgc
tnfn1_pw060510p03q177	FTN_0488	caacaaagattggcaaagtagta
tnfn1_pw060510p03q178	FTN_0898	aatttagtcgtcacagattcaac
tnfn1_pw060510p03q179	FTN_1079	tatttctgtaggtttcgtgactg
tnfn1_pw060510p03q180	FTN_0067	caaagttgctgttattactgagc
tnfn1_pw060510p03q181	FTN_1589	agaaggcgaaataattggtatag
tnfn1_pw060510p03q182	FTN_0125	caacttcgctagactatctcctt
tnfn1_pw060510p03q183	FTN_1264	gttggatggataaatgagagttt
tnfn1_pw060510p03q184	FTN_1715	tgtgaaactaaacctgcaatact
tnfn1_pw060510p03q185	FTN_1543	caggtgcatataagactattgga
tnfn1_pw060510p03q186	FTN_1417	ccagatggtcttagatgcaata
tnfn1_pw060510p03q187	FTN_0178	aacggttccatttcaaca
tnfn1_pw060510p03q188	FTN_0627	taatccaaatagtgttgcagatt
tnfn1_pw060510p03q189	FTN_0285	ctatattgttggctctgctagtg
tnfn1_pw060510p03q190	FTN_0371	tttgtaatgggctttatgatatg
tnfn1_pw060510p03q191	FTN_0973	acaaacttctccatgctcttc
tnfn1_pw060510p03q192	FTN_1098	tttcatcacagctataaataccg
tnfn1_pw060510p03q193	FTN_1513	taactatcaaagggatctattgc
tnfn1_pw060510p03q194	FTN_1628	atgtatcttatgtgcttcaatgg
tnfn1_pw060510p03q195	FTN_1243	agttaagtatagggatgcgctac
tnfn1_pw060510p03q196	FTN_0434	aaatatcgactctcttgctgata
tnfn1_pw060419p04q101	FTN_0829	ctagaacatcggcatagatttc
tnfn1_pw060419p04q102	FTN_1129	cttgaagagtcgccatactttat
tnfn1_pw060419p04q103	FTN_0183	gctgtgattggtttattatagcc
tnfn1_pw060419p04q104	FTN_0054	ataatcatctctcctggcatagt

tnfn1_pw060419p04q105	FTN_1683	aaagaagggtttgagatactttc
tnfn1_pw060419p04q106	FTN_1627	actcaaacatagtcacccaaga
tnfn1_pw060419p04q107	FTN_0022	gtcattattggtattggttgaaa
tnfn1_pw060419p04q108	FTN_1325	tgagtttggtagtgatagtgactt
tnfn1_pw060419p04q109	FTN_0910	tttagtcaagccacaagttacc
tnfn1_pw060419p04q110	FTN_0048	catgcttgtaaactaatgatgga
tnfn1_pw060419p04q111	FTN_0580	gcagctttgggatagtaaatatg
tnfn1_pw060419p04q112	FTN_1000	ataagcaacaatctccacagaat
tnfn1_pw060419p04q113	FTN_1435	tggtgcttcatctgtaaatgtat
tnfn1_pw060419p04q114	FTN_1361	tttgggtttattgctgttaatg
tnfn1_pw060419p04q115	FTN_0384	tttctatgatggtaaatgtggtc
tnfn1_pw060419p04q116	FTN_0287	atggcaaagaaattattatgagc
tnfn1_pw060419p04q117	FTN_1156	acaaatatgctgatgtgaaagag
tnfn1_pw060419p04q118	FTN_1172	tttatagtgatagccaaggtacg
tnfn1_pw060419p04q119	FTN_1024	caactgctcgtaatgctactaat
tnfn1_pw060419p04q120	FTN_0413	ctctatcgacatttactgctgag
tnfn1_pw060419p04q121	FTN_1104	tgaggtgtaatggtctaataatga
tnfn1_pw060419p04q122	FTN_1459	gcacgttcgctagataaactac
tnfn1_pw060419p04q123	FTN_0530	cattatcatccttagcgttgtag
tnfn1_pw060419p04q124	intergenic	cattagtttagtttgggttagattt
tnfn1_pw060419p04q125	FTN_1050	tgcagataaatataccctagcaa
tnfn1_pw060419p04q126	FTN_0296	atctccgatatgccagttc
tnfn1_pw060419p04q127	FTN_1342	agttatagacgcatggttcctt
tnfn1_pw060419p04q128	FTN_0679	tttagggtttatatgagctatcg
tnfn1_pw060419p04q129	FTN_1290	aagtgcttattatatccctttgc
tnfn1_pw060419p04q130	FTN_1585	cccaaaccagtaatttcatttat
tnfn1_pw060419p04q131	FTN_1744	acaactggcttacctcaaactat
tnfn1_pw060419p04q132	FTN_1282	aaagttgttcctgaaactcaaac
tnfn1_pw060419p04q134	FTN_0921	attcttggcttattaccattgat
tnfn1_pw060419p04q135	FTN_1415	cagcatggtactttagaattgag
tnfn1_pw060419p04q136	FTN_1601	tatgacatcttgacaaactttcg
tnfn1_pw060419p04q137	FTN_0289	tcctcagtatcaactttctcttg
tnfn1_pw060419p04q138	FTN_1470	tttgtatcgtttgtacttgcatt
tnfn1_pw060419p04q139	FTN_1109	tgagcttcaagtcctagatgata
tnfn1_pw060419p04q140	FTN_1465	catctgtatatgccgctaatct
tnfn1_pw060419p04q141	FTN_1118	tgctttgagttgccttgtatag
tnfn1_pw060419p04q142	FTN_0687	tactatcagggtgaaatgttagc
tnfn1_pw060419p04q143	FTN_0386	aactttagtatctccaaattcaataa
tnfn1_pw060419p04q144	FTN_0426	atcgcatattcataactttcg
tnfn1_pw060419p04q145	FTN_0901	ttgtaaatgctggtaatatgctt
tnfn1_pw060419p04q146	FTN_0961	aactaaatcatcatagccacca
tnfn1_pw060419p04q147	FTN_0334	tttacctgatgaagacaaactaaa
tnfn1_pw060419p04q148	intergenic	gcaatcttgtcttacttcatttg
tnfn1_pw060419p04q149	FTN_1502	aagcagcaaataccgtagataac
tnfn1_pw060419p04q150	FTN_0003	taaagtcataagaaaggcaacag
tnfn1_pw060419p04q151	FTN_1258	atcaaatagcaagactccataaa
tnfn1_pw060419p04q152	FTN_1017	tttagacatctgtatttactcacga
tnfn1_pw060419p04q153	FTN_0597	taggaccacaagatgctaataaa
tnfn1_pw060419p04q154	FTN_0550	ggtaggcttgaaataaatgaagt

tnfn1_pw060419p04q155	FTN_1654	tagcaaatgatacacctaaaccag
tnfn1_pw060419p04q156	FTN_0126	cttcgttggtatcatcagtattt
tnfn1_pw060419p04q157	FTN_0861	tttagtaggaatcataaagcagttt
tnfn1_pw060419p04q158	FTN_1430	aaacctttagctgccctcta
tnfn1_pw060419p04q159	FTN_0638	taggtattcaagttaaagcgtca
tnfn1_pw060419p04q160	FTN_0144	tttagtgataatcccaacgctat
tnfn1_pw060419p04q161	FTN_0085	atcagttggtacaagtagccttt
tnfn1_pw060419p04q162	FTN_1436	ataatagcaatacggtcaccttt
tnfn1_pw060419p04q163	FTN_1532	ctggtgatattggtgttggt
tnfn1_pw060419p04q164	FTN_0692	accacaacttctctatcaggatt
tnfn1_pw060419p04q165	intergnic	aatcagaagcaaagacataaaga
tnfn1_pw060419p04q166	FTN_0876	accctatttaattccatctcttg
tnfn1_pw060419p04q167	FTN_0620	agatagagcctaatagtgaacca
tnfn1_pw060419p04q168	FTN_1091	tcaaggttgtagtggagtatttg
tnfn1_pw060419p04q169	FTN_0761	tcagttactagagagccatttgt
tnfn1_pw060419p04q170	FTN_1762	gattgatctacataagccaaatg
tnfn1_pw060419p04q171	FTN_1252	ccacagcattattaggagaatta
tnfn1_pw060419p04q172	FTN_0757	tttacagaaacttattgccactg
tnfn1_pw060419p04q173	FTN_0868	tagtttcatcgcttctatattacca
tnfn1_pw060419p04q174	FTN_0272	caggaatcattgttctcactaat
tnfn1_pw060419p04q175	FTN_0973	catagacttggttagctgcatag
tnfn1_pw060419p04q176	FTN_1728	taaagcagaaagaccttgtatgt
tnfn1_pw060419p04q177	FTN_0595	ttagctgctgtttgatgatattt
tnfn1_pw060419p04q178	FTN_0028	caatgatgcactaaactttgact
tnfn1_pw060419p04q179	FTN_1001	tagtagcatctgtaagtggaagc
tnfn1_pw060419p04q180	FTN_1682	cttaaagcagcgcatacaat
tnfn1_pw060419p04q182	FTN_1716	tattgctaagtatttcgtggtca
tnfn1_pw060419p04q183	FTN_1240	aaagaaccagtttattgcgtta
tnfn1_pw060419p04q184	FTN_1540	gcttgtaaataagggcatatagtt
tnfn1_pw060419p04q185	FTN_1616	agtcaaagagcgaatcattt
tnfn1_pw060419p04q186	FTN_0745	taacaagtcgccaacaggta
tnfn1_pw060419p04q187	FTN_0777	aattatagaagaagagcgacgac
tnfn1_pw060419p04q188	FTN_0925	ataaatatgaaattggtgatcaaa
tnfn1_pw060419p04q189	intergenic	cagacataaccgcaacactg
tnfn1_pw060419p04q190	FTN_0391	tttatgcgcgttatatctttcta
tnfn1_pw060419p04q191	FTN_0077	actatttacttgggtttgtgcat
tnfn1_pw060419p04q192	FTN_1427	tacatgataagccagagagacc
tnfn1_pw060419p04q193	FTN_1291	tgagtgctttatactgttatttgc
tnfn1_pw060419p04q194	FTN_0771	agcttcaacactatcatcatctt
tnfn1_pw060419p04q195	FTN_0121	gcttcttgatctcctaatctcat
tnfn1_pw060419p04q196	FTN_1088	aaattggaaagctaatggttg
universal primer	n/a	tcagaattggttaattggttgt
