# Supplementary material for: Restriction of Francisella novicida Genetic Diversity during Infection of the Vector Midgut
Source: PLoS Pathog. 2014 Nov 13;10(11):e1004499. doi: 10.1371/journal.ppat.1004499 (PMC4231110; doi:10.1371/journal.ppat.1004499)
Supplement: Table S3 — Parameters used in the standard runs of the model for vectors, hosts, and pathogens, and values used in the sensitivity analyses of vector-to-host ratios, vector and host abundance, and initial pathogen genotypic diversity. (DOCX) [file ppat.1004499.s009.docx]

| Model Parameter | Standard Value | Values in sensitivity analysis |
| --- | --- | --- |
| Number of mice | 100 | 10, 50, 100, 500, 1000 |
| Number of ticks | 1000 | 100, 500, 1000, 5000, 10000 |
| Number of pathogen genotypes | 100 | 10, 50, 100 |
| Vector-to-host ratio | 10:01 | 1:1, 5:1, 10:1, 50:1, 100:1 |

Table S3. Parameters used in the standard runs of the model for vectors, hosts, and pathogens, and values used in the sensitivity analyses of vector-to-host ratios, vector and host abundance, and initial pathogen genotypic diversity.
