# Supplementary material for: Restriction of Francisella novicida Genetic Diversity during Infection of the Vector Midgut
Source: PLoS Pathog. 2014 Nov 13;10(11):e1004499. doi: 10.1371/journal.ppat.1004499 (PMC4231110; doi:10.1371/journal.ppat.1004499)
Supplement: Table S4 — List of genotypes, recovered from mice but not ticks in pooled genotype experiments, that were further investigated in single-infection assays and 1∶1 competition assays with wild-type. (DOCX) [file ppat.1004499.s010.docx]

Table S4. List of genotypes, recovered from mice but not ticks in pooled genotype experiments, that were further investigated in single-infection assays and 1:1 competition assays with wild-type.

|  | locus tag | gene name | description |
| --- | --- | --- | --- |
| Genotype 1 | FTN_1356 | *recD* | exodeoxyribonuclease V, alpha subunit |
| Genotype 2 | FTN_0787 | *rep* | UvrD/REP superfamily I DNA and RNA helicases |
| Genotype 3 | FTN_1417 | *manB* | phosphomannomutase |
| Genotype 4 | FTN_0530 | *mpl* | UDP-N-acetylmuramate:L-alanyl-gamma-D-glutamyl-meso-diaminopimelate ligase |
| Genotype 5 | FTN_1109 | - | rhodanese-like family protein |
| Genotype 6 | FTN_1682 | *frgA* | siderophore biosynthesis protein |
